# Increasing effort without noticing: A randomized controlled pilot study about the ergogenic placebo effect in endurance athletes and the role of supplement salience

**DOI:** 10.1371/journal.pone.0198388

**Published:** 2018-06-11

**Authors:** Ellen K. Broelz, Sebastian Wolf, Patrick Schneeweiss, Andreas M. Niess, Paul Enck, Katja Weimer

**Affiliations:** 1 Department of Psychosomatic Medicine and Psychotherapy, University Hospital Tübingen, Tübingen, Germany; 2 Department of Psychology, University of Tübingen, Tübingen, Germany; 3 Department of Sports Medicine, University Hospital Tübingen, Tübingen, Germany; 4 Clinic for Psychosomatic Medicine and Psychotherapy, University Hospital Ulm, Ulm, Germany; University of Bourgogne France Comté, FRANCE

## Abstract

**Purpose:**

Previous research shows that endurance performance can be enhanced by placebo ergogenic aids. This study investigates the ergogenic placebo response, which we define as an increase in objective and physiological effort without an increase in subjective effort, in competitive cyclists. The primary objective of this study is to explore the role of supplement salience in the ergogenic placebo response, while the secondary aim is to assess whether believing to have taken an inactive placebo supplement attenuates the desired ergogenic effect.

**Methods:**

We employed a double-blind placebo-controlled study design and compared a high salience (pudding) to a low salience (capsules) ergogenic placebo supplement and to a no treatment control group. Thirty-four male athletes (30.0 ± 5.7 years) performed two self-regulated time trials on an isokinetic cycling ergometer, one without intervention serving as a baseline and one with intervention according to group assignment. At both time trials, power output (objective effort), blood lactate (physiological effort) and the rating of perceived exertion (subjective effort) were measured.

**Results:**

Receiving a high salience supplement can increase physiological and objective effort without a proportional rise in subjective effort, suggesting a decoupling of perceived exertion and endurance performance. Low salience and control group both showed no such ergogenic placebo response. Athletes’ belief concerning the true nature of the ergogenic aid (inactive placebo vs. ergogenic supplement) did not influence the ergogenic placebo response.

**Conclusion:**

High salience placebo ergogenic aids can elicit enhanced performance without the athlete noticing (exertion), and deception of athletes seems unnecessary as even believing to have received an inactive placebo supplement maintains the ergogenic placebo response.

## 1. Introduction

More than 80% of competitive athletes consume dietary supplements as ergogenic aids for performance enhancement on a daily basis [1–3]. However, previous research has shown that exercise performance can be influenced not only by the ingredients of supplements but also by expectancy effects caused by beliefs about the efficacy of such ergogenic aids [4–6]. These expectancy effects are commonly known as the placebo effect, which is defined as any change in physical or physiological condition following an inert treatment [7]. However, it is unclear if characteristics of ergogenic aids affect the perception of their efficacy and, therefore, the elicited placebo effect as it has been shown in the medical context [8,9].

In the past two decades, a number of researchers have sought to determine the role of the placebo effect in physical performance. In 2008, Pollo et al. showed that placebo-induced expectations of better performance can increase muscle work by reducing fatigue and exertion perception, suggesting top-down modulation by altered sensory processing [10,11]. Meta-analyses examining the placebo effects in various strength and endurance sports disciplines including power lifting, cycling and running found a medium effect size for overall performance enhancement [12] and placebo responses between -7.8% and +50.7% [13].

The role of ergogenic aid salience has not been addressed explicitly in the research of the ergogenic placebo effect in terms of sports performance. However, several studies examined the placebo response of specific dietary supplements administered in different forms and thus of varying degrees of salience in medical settings. These studies individually assessed the ergogenic placebo response to capsules [14] or drinks [15,16] and even discussed the influence of preparation form and color on patients’ expectations [17]. Further, the association of physiological change and salient flavors was shown in classical behavioral conditioning of immune responses [18,19] and of motion sickness severity [20]. These studies showed, that immune responses can be classically conditioned using a salient novel tasting drink (green strawberry milk with lavender scent), and taste aversion to a salient pleasantly flavored drink (elderberry juice) can be created by pairing it with the sensation of motion sickness. Campbell and colleagues indirectly addressed ergogenic aid salience by comparing the efficacy of carbohydrate supplements in form of sport beans, sport drink or gel, yet found no difference in metabolic response and exercise performance [21]. Further, according to a questionnaire based study of 267 college students, shape (liquid, lotion, pill, capsule, bar, powder), color and mode of administration (drink, swallow, skin application) can influence the perceived effectiveness of ergogenic aids with regard to enhancing strength, endurance and concentration at least in theory [22]. Overall, these studies show that the evidence regarding the influence of supplement salience on their ergogenicity is still inconclusive.

It has been shown that the placebo response is influenced by cognitive factors such as the patients’ expectations regarding the efficacy of the given treatment [7,23] also in the sports specific context [24,25]. Treatment expectations in double-blind placebo-controlled studies can be assessed by recording whether subjects believe to have received an active treatment or an inactive placebo. For the purpose of this study, we define the ergogenic placebo response (EPR) as an increase in objective effort (*OE*) [power output in watt], and an increase in physiological effort (*PE*) [blood lactate in mmol/l], without an increase in subjective effort (*SE*) [Rating of Perceived Exertion (RPE) scale] [26] as cyclists were instructed to show their highest performance. This definition is in line with previous research outcomes [27,28].

We employed a classical double-blind placebo-controlled study design with a no treatment control group, in which all participants were informed, that they had a 50% chance of receiving either a potentially performance enhancing BCAA supplement or a placebo, or will be randomized to be in the no treatment control group. The primary aim of this study was to investigate the influence of salience in the EPR in endurance performance in competitive cyclists. Therefore, we designed a novel and highly salient ergogenic placebo supplement in form of a food product (pudding), stimulating various senses: gustation (bitter-sweet grapefruit flavor), olfaction (vanilla odor), vision (pink color) and lastly oral tactition (semi-solid texture) and mechanoreception (using spoon for ingestion). The group receiving the high salience (*HS*) placebo supplement was compared to a group receiving white capsules, as a low salience (*LS*) contrast and to the control group (*C*). The secondary objective of this study was to explore whether believing to have taken an inactive placebo compared to an active supplement attenuates the ergogenic placebo effect. To quantify the placebo effect, athletes performed two self-regulated time trials (TT): baseline (TT_b_) and intervention (TT_i_). Both time trials had a known end point and physiological and psychological measures were taken.

We formulated the following hypotheses: (1) Both placebo (*HS* and *LS*) groups show an ergogenic placebo response, while the control group does not. Further, high salience enhances the EPR, meaning that we expect an interaction of time and group. Specifically, we expect a larger increase in *OE* and *PE* in the high salience group compared to control and compared to *LS* groups. As athletes are instructed and are in general motivated to show their maximal performance, decoupling of *SE* and *PE* could be possible, and we hypothesize that the ergogenic placebo response enables higher performance without changes in *SE*. (2) Furthermore, we expect the ergogenic placebo response to be more pronounced in those athletes, who believed to have received an ergogenic aid compared to those who believed to have received an inactive placebo.

## 2. Materials & methods

Methods are based on a previously published study protocol [29], with minor changes in data acquisition and consequently data analysis.

### 2.1 Participants

**Fig 1:**
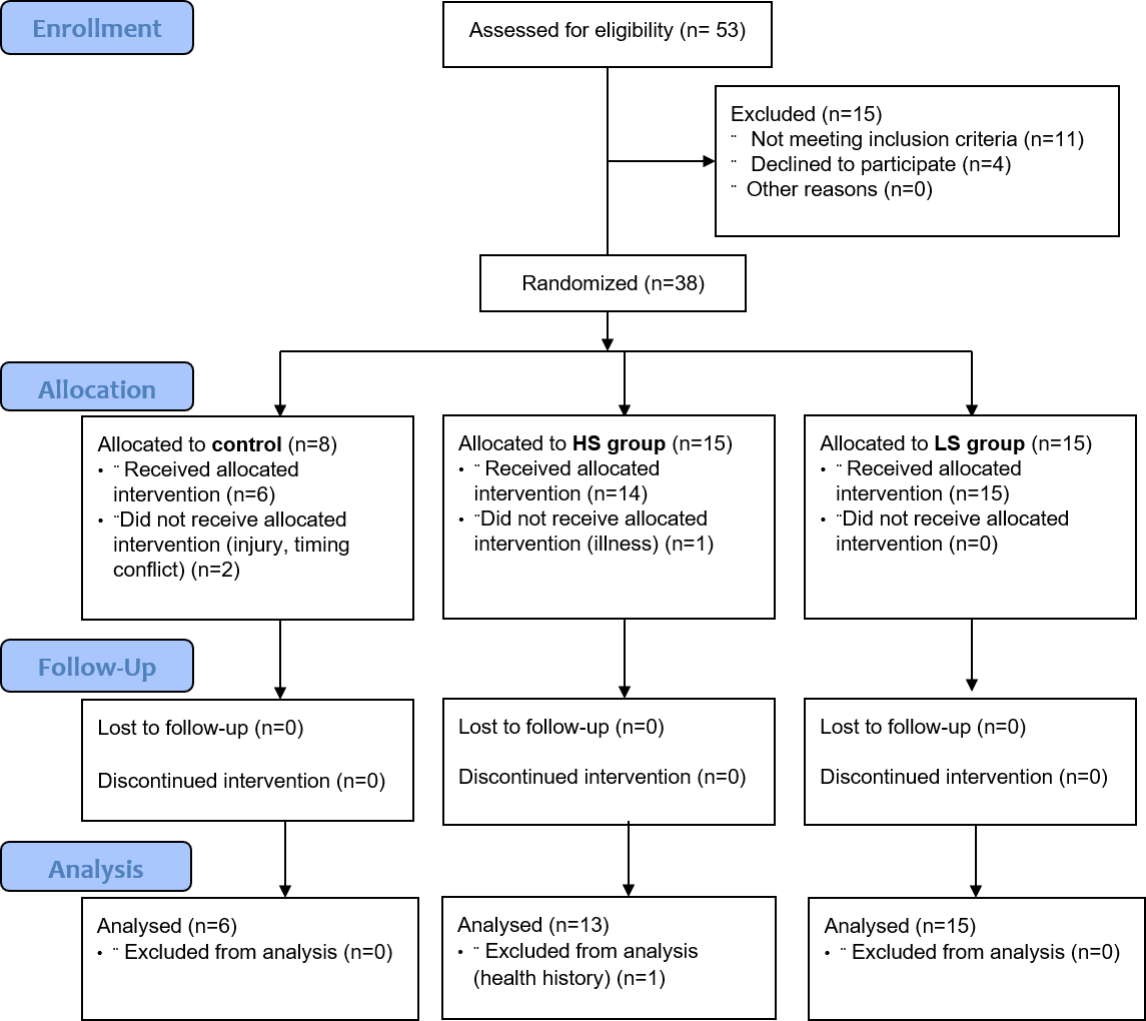
Group assignment. HS = high salience, LS = low salience, C = control.

An a priori power analysis (G*Power Version 3.1.9.2.) for 3 (group) x 2 (time) within-between factors repeated measures ANOVAs with an assumed medium effect size of Cohen’s f = .3 (power = .80, alpha = .05, two-tailed) for all three main measures (OE, PE, SE) revealed a required sample size of N = 30 participants. Taking possible drop-outs into account, we recruited 38 cyclists, of which two dropped out due to injury and timing conflicts, one was excluded due to his health history and one because of a flu in the week between time trials. All participants had to fulfill the following inclusion criteria: male, between 18 and 45 years of age, a minimum of 3 weekly training sessions on the bicycle during racing season and a minimum of 3 competitions per year (Fig 1). Exclusion criteria were performance impairing chronic illness and a training pause exceeding 7 consecutive days in the 4 weeks immediately prior to the study due to illness or injury. Previous experience with the use of supplements was equal between athletes and no previous experience with BCAA supplementation was reported. All data acquisition took place at the performance diagnostics laboratory at the Department of Sports Medicine at the University Hospital in Tuebingen, Germany. The study was approved by the ethical committee of the University of Tuebingen and registered at the German Registry for Clinical Trials (DRKS, DRKS00005802). All participants were enrolled after written informed consent only.

### 2.2 Study design

Athletes visited the performance laboratory on four separate occasions: (1) performance test to determine current training level, (2) test time trial (TT_t_) to become familiar with the isokinetic ergometer, (3) baseline time trial (TT_b_), and (4) intervention time trial (TT_i_). The first block of data collection lasted from February until July 2014 and the second block lasted from May until June 2015.

**Fig 2:**
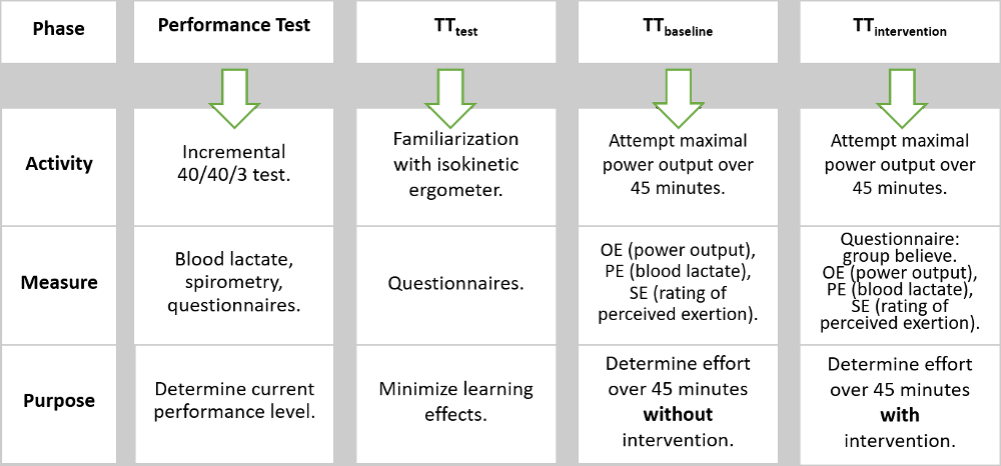
Overview of experimental procedure. OE, objective effort; PE, physiological effort; SE, subjective effort.

Both TT_b_ and TT_i_ were scheduled at the same time of day (8 am or 10 am) and the same day of the week (Wednesday, Thursday or Friday) spaced 7 days apart in order to minimize performance changes due to influences of daytime and weekly training differences. Before TT_i_, participants were assigned to one of three groups via block randomization: Low Salience (*LS*) (n = 15), High Salience (n = 13) or no treatment control (*C*) (n = 6) group. Enrichment randomization was applied in order to maximize the number of participants in the intervention groups, while controlling for performance changes due to learning and training effect with a control group. As this was a double-blind study, a laboratory assistant selected interventions according to a randomization list kept in a closed envelope. Based on athletes’ responses on the post-intervention questionnaire, given immediately after intervention and before the start of the time trial, sub-groups were formed based on group belief: believed to have received ergogenic supplement (*ES*) vs. placebo (*P*). Fig 2 provides a graphical overview of the experimental procedure.

### 2.3 Intervention

Due to the large amount of information about ergogenic supplements and the high degree of experimentation and experience with them among competitive athletes, we created a novel yet plausible cover story. This was necessary to keep the factors of knowledge and experience with the ergogenic aid constant among athletes and enable us to measure the influence of salience. Further, we aimed to avoid deception of the athletes by employing a double-blind placebo-controlled study design using an ineffective low dose of branched chain amino acids (BCAA) as an "active" placebo compared to an inert placebo. While inert placebos are devoid of physiological action, active placebos have a physiological action, which is not necessarily relevant to the task [6] or is given to mimic side effects [30]. Although we use BCAA not to mimic side effects, as the original use of the term suggests [31], we find the term active placebo appropriate in this study of the placebo effect, because we use it to enhance expectations and avoid differences regarding athletes’ experience with the supplement as well as induce high expectation by providing scientific evidence for their possible ergogenic potential in higher doses [32–36]. The intake of high doses of the BCAAs leucin, valine and isoleucine during endurance exercise has been hypothesized to decrease fatigue perception by competing with tryptophan, a neurotransmitter precursor of serotonin (5-HTP), at the blood brain barrier [37].

The *HS* groups received a vanilla-grapefruit flavored pudding (6g corn starch, 15g erythritol, 200mg vanillin, red food color, 75ml H_2_O) with either 4.3g BCAA (1.5g Valin, 1.3g Leucine, 1.5g Isoleucine) as active placebo or 1 ml of 15.3 mmolar chinin sulphate solution as inert placebo. Chinin sulphate was used to match the bitter flavor of BCAA. We created the pudding for high stimulus salience: gustation (bitter-sweet grapefruit), olfaction (vanilla), vision (pink color), oral tactition (semi-solid) and mechanoreception (spoon for ingestion). The *LS* groups received 8 white capsules size 00, summing up to 4.3g BCAA for the active placebo or 6g cornstarch for the inert placebo. The carbohydrate content in form of cornstarch is low enough to be certain of no physiological effect on muscle glycogen content [38]. BCAA, chinin sulphate and vanillin were obtained from Fagron GmbH & Co. KG (Barsbüttel, Germany).

### 2.4 Performance test

After athletes read the study description and agreed to participate, a medical doctor examined their resting electrocardiogram (ECG), heart and lung function and cleared them for participation in the study. We primed the right ear lobe with Finalgon® crème (4 mg/g + 25 mg/g Nonivamid and Nicoboxil) (Boehringer Ingelheim Pharma GmbH & Co. KG) to increase blood circulation and facilitate lactate sampling. Inhaled and exhaled air were measured via spiro-ergometry to determine oxygen consumption (VO2), carbon dioxide production (VCO2) and total ventilation throughout the incremental performance test as well as ECG, starting at 40 Watt and increasing by 40 Watt every 3 minutes. During this protocol, athletes cycled continuously until they were no longer able to maintain cadence above 65 rpm. Blood samples (20μl) were taken from the right earlobe at rest and at the end of each 3 min interval to determine lactate concentration at each workload. Blood samples were analyzed in a stationary blood lactate analyzer (Biosen S_line by EKF Diagnostics, Barleben, Germany). The lactate threshold (LT) and the individual anaerobic threshold (IAT) were calculated with the Dickhuth method [39]. From the spiro-ergometry data we were able to determine the maximal oxygen uptake (VO2 max).

### 2.5 Time trials

Throughout all time trials, athletes were able to monitor their power output and heart rate, which was transmitted via a chest strap pulse sensor (Polar Electro GmbH, Büttelborn, Germany) on a small display on the handlebar of the ergometer (SRM GmbH, Jülich, Germany). Every session began with a 10 min warm up at a resistance of 1.5 W/kg body weight (BW) and ended with a 5 min cool down at varying cadence and a resistance of their choice. During the time trial, cadence was fixed at 95 rpm in the isokinetic mode. Participants were instructed to go all out at each time trial and try for maximal power output over time. Blood lactate and RPE were measured at rest and in 5 min intervals throughout the time trial.

TT_t_ (30 min test time trial): Athletes were instructed to use this test session to become familiar with the isokinetic properties of the ergometer.

TT_b_ (45 min baseline time trial): Athletes drank 250 ml of water 20min before the beginning and 250ml during the time trial.

TT_i_ (45 min intervention time trial): All subjects received an information sheet designed to increase expectancy associated with acute BCAA ingestion on high-intensity endurance capacity, emphasizing its potential ergogenic properties by way of reducing fatigue and exertion perception.

The *LS* groups received 250ml water to swallow eight capsules, the *HS* groups received 150ml water to wash down 100ml of pudding eaten with a spoon, and the control group received 250ml water. The precise amount of water was given to ensure similar stomach volume between groups. Immediately after receiving the intervention, athletes received a questionnaire asking which group they believed to be in (placebo or ergogenic supplement). During the time trial, all athletes were allowed to drink 250ml of water. Twenty minutes after the intervention, the time trial began.

### 2.6 Data analysis

Objective effort (*OE*) was determined by averaging power output over 45 minutes and normalized for current training level by subtracting IAT. Physiological effort (*PE*) was determined by subtracting the blood lactate value after warm up from the mean lactate of the 45 min time trial. Subjective effort (*SE*) was determined by subtracting the rating of perceived exertion (RPE) after warm up from the mean RPE of the 45 min time trial. All values were determined for both time trials, TT_i_ (time trial at intervention) and TT_b_ (time trial at baseline).

For the manipulation check comparing inert and active placebos we used an independent-samples equivalence procedure based on the Cohen's classification of effect sizes [40].

For analyses of main results, we compared the change from TT_b_ to TT_i_ between groups (*HS*, *LS*, *C*) for all three main measures (*OE*, *PE*, *SE*) using 3x2 repeated-measures ANOVAs after testing for normality using the Shapiro-Wilk test. For the sub-group analysis (group belief ergogenic supplement (*ES*) vs. placebo (*P*)) we used 2x2 repeated-measures ANOVAs. Post-hoc independent and paired t-tests were carried out when appropriate. Statistical significance was determined a priori at p < .05, and p-values between .05 and .10 were rated as marginally significant due to the exploratory nature of this study. All data was analyzed using SPSS version 19.0 (IBM SPSS Statistics for Windows, Armonk, NY, USA).

## 3. Results

### 3.1 Sample characteristics

Thirty-four male athletes between 18 and 45 years (30.0 ± 5.7 years) were included in data analysis (Table 1).

**Table 1:** Sample characteristics. Descriptive statistics are presented as mean value ± standard deviation.

	Control (C)	High Salience (HS)	Low Salience (LS)
No. of subjects	6	13	15
Age (years)	30.33 ± 3.39	29.46 ± 6.44	30.33 ± 6.04
Weight (kg)	71.25 ± 3.84	75.85 ± 4.70	73.33 ± 8.94
Height (m)	1.81 ± 0.06	1.81 ± 0.06	1.79 ± 0.06
BMI (kg/m^2^)	21.84 ± 1.73	23.14 ± 1.62	22.87 ± 2.37
IAT (watt)	233.17 ± 37.86	244.08 ± 24.53	240.33 ± 35.52
VO_2_ Max (ml/min/kg)	57.67 ± 6.59	57.62 ± 4.23	57.47 ± 6.39

### 3.2 Manipulation check

To assure the assumption of equivalence of low dose BCAAs as active placebos compared to inert placebos in terms of their ergogenic efficacy, independent of the form of administration, we used an independent-samples equivalence procedure assuming a Cohen’s ∆ of .5. The test showed statistical significant equivalence between inert and active placebo on all three main measures. *OE* (p < .05), *PE* (p < .05) and *SE* (p < .05). Furthermore, the amount of participants in the LS and HS groups who expected to be in the ergogenic supplement and the placebo group did not differ (Χ^2^ = .26, p = .61).

### 3.3 Effects of salience on physiological and objective effort

**Fig 3:**
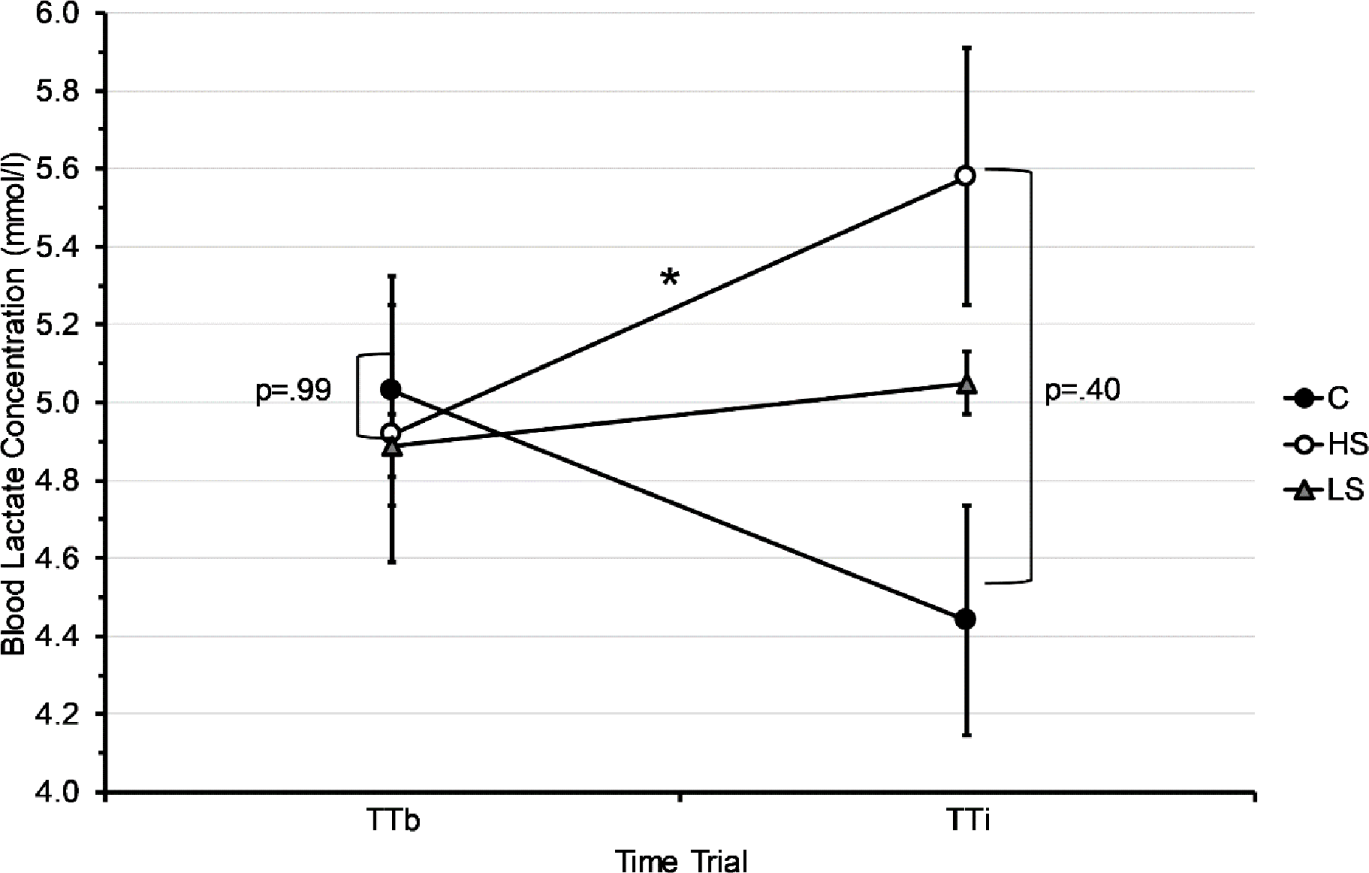
Interaction of time (TT_b_ vs. TT_i_) and group (*HS*, *LS*, *C*) on physiological effort presented as mean blood lactate (mmol/l) over 45 minutes above post warm up blood lactate. * p < .05.

The Shapiro-Wilk test of normality showed normal distribution for all groups (*HS*, *LS*, *C*) on all three main measures (*OE*, *PE*, *SE*) at both TT_b_ and TT_i_. To investigate the influence of salience on physiological effort, we conducted a repeated measures ANOVA of group (*HS x LS x C*) and time (TT_b_ x TT_i_) on *PE*, which revealed a marginally significant interaction of time and group (F(2,31) = 3.26, p = .05, partial η^2^ = .17). Despite this marginally significant interaction we performed exploratory post-hoc t-tests, which revealed a significant increase in blood lactate from TT_b_ to TT_i_ in the *HS* group (from M = 4.92 ± SD = 1.60 to M = 5.58 ± SD = 1.96; t(12) = -2.66, p = .02), but no significant changes in the *LS* (t(14) = -.60, p = .56) and control group (t(5) = 1.28, p = .26) (Fig 3).

A repeated measures ANOVA of group (*HS x LS x C*) and time (TT_b_ x TT_i_) for objective effort revealed a significant main effect for time (F(1,31) = 18.71, p < .001, partial η^2^ = .38), but no interaction of time and group (F(2,31) = 1.58, p = .22, partial η^2^ = .09).

**Fig 4:**
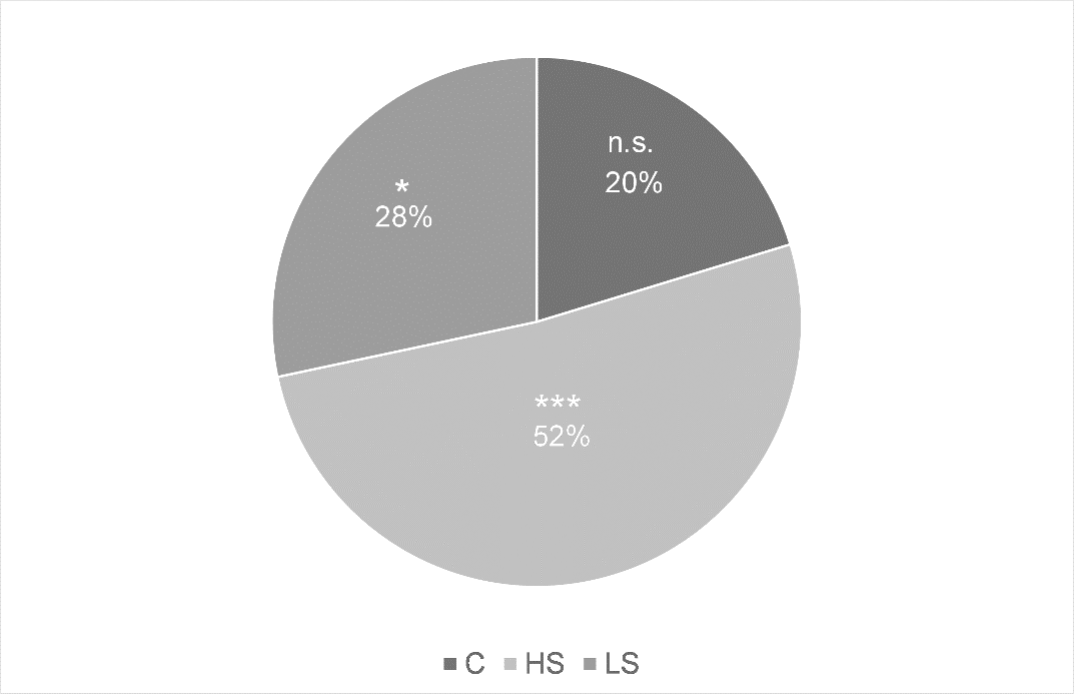
Change in objective effort (OE) from TT_b_ to TT_i_ given in percent change. * p < .05, *** p < .001.

For further exploratory data analysis, we conducted post-hoc t-tests which revealed no significant differences between groups at TT_b_, and between control group and each intervention group at TT_i_ (all p’s > .10). There was a marginally significant difference between HS and LS groups at TT_i_ (t(26) = 1.98, p = .06) with higher power output in the HS compared to the LS group (28.60 ± 21.03 vs. 12.15 ± 22.62 Watt above IAT). Paired t-tests for the change between TT_b_ and TT_i_ revealed significant increases in power output for the HS group (t(12) = -4.92, p < .001) as well as for the LS group (t(14) = -2.25, p = .04), but no change in the control group (t(5) = -2.03, p = .10) (Fig 4).

### 3.4 Effects of salience on subjective effort

To investigate our hypothesis, that there is no difference in subjective effort between time trials across groups, we conducted a repeated measures analysis of variance (ANOVA) with the factors group (*HS x LS x C*) and time (TT_b_ x TT_i_). Indeed, there was no significant main effect of time (F(1,31) = .23, p = .64 partial η^2^ = .01) or interaction of time and group (F(2,31) = 1.68, p = .20, partial η^2^ = .1) for *SE*.

### 3.5 Effects of stimulus expectancy on outcomes

For hypothesis two, we conducted three repeated measures ANOVAs to compare the effect of group belief (BCAA: N = 18 vs. placebo: N = 10) on *OE*, *PE* and *SE*. The results revealed no significant interaction of group belief and time for *OE* (F(1,26) = .01, p = .94, partial η^2^ = .00), *PE* (F(1,26) = .54, p = .47, partial η^2^ = .02) and *SE* (F(1,26) = .72, p = .41, partial η^2^ = .03). A main effect of time was observed for *OE* only (F(1,26) = 18.64, p < .001, partial η^2^ = .42). Post-hoc tests showed a significant increase in *OE* in all athletes: In those who believed they received the ergogenic supplement (t(17) = -3.50, p<0.01) as well as in those who believed to have received an inactive placebo (t(9) = -2.94, p<0.01).

## 4. Discussion

Researchers have argued that exercise performance is always submaximal, as athletes always stop in time to prevent metabolic catastrophes or cardiorespiratory failure [41]. A physiological ‘‘reserve” capacity will therefore always remain and be available to mobilize using performance enhancing (ergogenic) placebos. Therefore, this study set out with the aim of investigating the ergogenic placebo effect in endurance athletes and assessing the role of supplement salience by comparing the form of administration as a novel highly salient food, stimulating a multitude of senses, with the traditional administration in form of low salient plane white capsules. The aim was to gain insights in how to mobilize the above-mentioned reserves in order to enhance performance in a healthy, non-harmful and naturally doping-free manner.

As expected, the data shows an interaction of group and time regarding physiological effort (*PE)*. Specifically, *PE* increases significantly from TT_b_ to TT_i_ in the *HS* group, while there is no significant change in the *LS* and the control group (Fig 3). Contrary to our hypothesis, however, this study did not find a significant interaction of group and time in objective effort (*OE)*. Due to the exploratory nature of this study and the specificity of our hypothesis, that an ergogenic aid causes a placebo response compared to a control group, we carried out further data analysis. This exploratory analysis indicated significant increases in *OE* from baseline to intervention in the *HS* as well as in the *LS* group, but not in the control group. These results suggest, that receiving a highly salient ergogenic aid increases *PE* more than receiving a low salience or no supplement. Exploratory analyses suggest, however, that salience does not affect the degree of change in *OE*.

As hypothesized, we found increases in physiological and objective efforts due to an ergogenic placebo without a rise in subjective efforts. Our data is therefore in line with previous research: A recent publication reporting improved exercise performance without change in perceived effort following administration of a placebo, suggests a “degree of decoupling in the normal relationship between RPE and exercise intensity” (p. 1679) as the explanation [27]. Further, Williams et al. suggest that an increase in performance without increases in perceptions of exertion may be due to reduced focus on afferent sensory feedback [28]. Overall, this study provides evidence for the notion that the clue of the ergogenic placebo response is that it allows athletes to increase performance because they do not perceive it to be as strenuous as they would without the EPR and this effect seems to be enhanced by salience.

Further, recent research suggests an association of taste [42] and nutrient receptor activation [43] and exercise performance, as has been shown in mouth rinsing studies in the endurance [44] and the strength domain [45]. While we can assume, that the pudding used as a salient ergogenic aid in this study stimulated both bitter (quinine / BCAA) and sweet (erythritol) taste receptors, as well as CHO receptors (cornstarch), the capsules, which were used as a low salience ergogenic aid, elude this receptor stimulation. Mouth sensing may thus play a role in the placebo response observed in the *HS* and not in the *LS* group in this study.

Our data shows an EPR for the group receiving a highly salient placebo supplement (increased *PE*, increased *OE*, unchanged *SE*) and partially for the group receiving a low salient one (unchanged *PE*, increased *OE*, unchanged *SE*). This result is consistent with our prediction, as we expected both placebo groups to show an EPR, albeit a smaller one in the *LS* group. The exploratory post-hoc tests showed a smaller but significant increase in *OE* in the *LS* than in the *HS* group. Our hypothesis was based on the assumption that capsules are inadvertently classically conditioned stimuli, where learning occurred simply through repeated coupling of capsules with physical (e.g. pain medication–pain reduction) or physiological (e.g. antihistamine–allergy symptom reduction) consequences throughout life. Research has shown repeatedly, that conditioning plays a key role in the placebo effect [7]. A possible explanation for the overall low placebo response may be that athletes were instructed to maximize power output during the time trials and attempt to achieve maximal effort. Pushing oneself in a laboratory setting in the absence of extrinsic motivation in the form of training partners and competitors is not only artificial, as cycling athletes rarely train alone, it requires a lot more self-motivation than cycling with an opponent [46]. These characteristics of the laboratory setting are disadvantageous for reaching optimal performance and given real life circumstances, we expect the EPR could be larger and unleash even more of athletes’ hidden reserves.

Surprisingly, we did not find a significant difference between those athletes who believed they had received the ergogenic supplement and those who believed they had received an inactive placebo regarding the change in *OE*, *PE* and *SE* from baseline to intervention. Thus, it seems that the placebo response is not dependent on whether the athlete explicitly believes to have taken an ergogenic substance. This finding is highly relevant for the ethical debate surrounding the deceptive administration of placebos [47,48]. Studies testing the non-blind administration of placebo date back to the 60s [49] and more recent findings confirm the effectiveness of open-label placebo administration in treating irritable bowel syndrome [50], migraine attacks [51], ADHD [52], depression [53,54] and in analgesia [55]. The present study suggests, that even in the field of exercise performance enhancement, informing the athlete about receiving a placebo will likely maintain the ergogenic placebo response.

Our study has several strengths, displaying the ergogenic placebo response in competitive athletes and comparing low and high salience supplement characteristics. However, some limitations should be noted. First, the overall sample size is small, although group sizes are comparable to or even larger than previous studies of this sort [10,56,57]. Second, the results may be confounded by intra-individual variability in factors such as different quality of sleep, diet and hydration level at baseline and intervention time trial, which we attempted to minimize by running both time trials at the same time of day and the same day of the week. Inter-individual differences in the absorption of BCAA and thus possible differences in their influences on metabolism were controlled via statistical equivalence testing only and not via blood tests. Finally, a more realistic environment such as a competitive multiple athlete race setting could impact the results.

## 5. Conclusions

We conclude that ergogenic placebos have the potential of increasing power output and muscle work without the expected proportional increase in effort perception and that this ergogenic effect is enhanced by increased supplement salience. Whether participants believed to have received an ergogenic supplement or an inactive placebo did not change the placebo response substantially.

On a practical level, this study advocates a more holistic approach to performance in competitive athletes and underscores the important role of the ergogenic placebo response and the role of supplement salience, beliefs and expectation on exercise performance. These results are relevant for both the treatment of and the interaction with athletes in the context of sports medicine, physical therapy and nutritional interventions. It shows that open discourse about the use of placebos is possible and suggests that coaches and athletes could use this knowledge of the ergogenic placebo effect to augment treatment responses and allow for better-informed decisions regarding the use of performance enhancing products. Further studies investigating the central mechanisms underlying placebo-induced changes in performance may elucidate the nature of the decoupling between subjective and objective effort. Future research should enhance our understanding of the neurobiological basis of factors limiting and enhancing physical performance.

## Supporting information

S1 File CONSORT checklist. (DOC)

S2 File Translation of the study protocol (English). (DOCX)

S3 File Original study protocol (German). (DOCX)

S4 File Translation of the participant information and consent form (English). (DOCX)

S5 File Original participant information and consent form (German). (DOCX)
