# Supplementary material for: Increasing effort without noticing: A randomized controlled pilot study about the ergogenic placebo effect in endurance athletes and the role of supplement salience
Source: PLoS One. 2018 Jun 11;13(6):e0198388. doi: 10.1371/journal.pone.0198388 (PMC5995445; doi:10.1371/journal.pone.0198388)
Supplement: S2 File — (DOCX) [file pone.0198388.s002.docx]

**„Acute effect of a nutritional supplement on endurance performance – a placebo controlled double blind study“**

Study execution led by:

Prof. Dr. med. A. Nieß

Medical Director – Department of Sports Medicine

University Hospital Tübingen

Hoppe-Seyler-Str. 6

72076 Tübingen

Tel.: [07071-29 86493](tel:07071-29%2086493)

Fax: [07071-29 25028](tel:07071-29%2025028)

e-mail: [andreas.niess@med.uni-tuebingen.de](mailto:andreas.niess@med.uni-tuebingen.de)

<http://www.med.uni-tuebingen.de/sportmedizin/index.htm>

Involved researcher:

Ellen Kristina Brölz, MSc

Funding:

The study will be financed by departmental funds. External research funding will be acquired by the Danone Stiftung and the Else-Kröner Fresenius Stiftung.

____________________________

Prof. Dr. med. A. Nieß

**Summary**

Performance enhancement is a goal not only for professional athletes, but also for competitive athletes and performance oriented hobby athletes. If training itself is already optimized, other options to enhance performance are searched for. Many athletes want to optimize their diet for this reason and therefore use specific foods or food supplements, which they know or belief to influence their performance, recovery or overall wellbeing.

In this context amino acids, specifically branched chain amino acids (BCAA), play a key role. Their supporting function in recovery are well researched, while studies looking at their performance enhancing effect are lacking. This study attempts to close this research gap.

This is a double blind randomized placebo controlled trial to investigate the acute effect of a BCAA supplement on endurance performance in healthy endurance athletes training in the discipline of cycling, running or triathlon. In the experiment, the BCAAs and the placebos will be administered either in the form of capsules (supplement) or pudding (food). Each participant will be tested three times. In the pretest session, the individual maximal oxygen uptake will be determined. Performance will be measured at the first test session without an intervention and at the second session after administration of a BCAA or placebo intervention. Session one and two will be spaced two days apart.

Subjects are informed, that they are participating in a randomized double-blind trial, in which they will either receive a nutritional supplement or a placebo. Besides primary endpoints (subjective and objective endurance performance), expectation regarding the intake of a BCAA supplement will be measured. Before and after the spiro-ergonomic performance tests, all participants will receive psychometric questionnaires.

**Table of contents**

[1. List of abbreviations used in the test protocol 4](#_Toc484524107)

[2. Background 4](#_Toc484524108)

[2.1 State of the art 4](#_Toc484524109)

[2.1.1 Nutritional supplement in competitive sports 4](#_Toc484524110)

[2.1.2 Nutritional supplements vs. functional food 5](#_Toc484524111)

[2.1.3 Central and peripheral fatigue and the roll of BCAAs 6](#_Toc484524112)

[2.1.3 Food and the placebo effect 7](#_Toc484524113)

[2.2 Study purpose 8](#_Toc484524114)

[3. Study goals 8](#_Toc484524115)

[4. Duration 9](#_Toc484524116)

[5. Study population 9](#_Toc484524117)

[5.1 Description of study population 9](#_Toc484524118)

[5.2 Inclusion and Exclusion criteria 9](#_Toc484524119)

[5.3 Recruiting 10](#_Toc484524120)

[5.4 Number of participants 10](#_Toc484524121)

[6. Study protocol and experimental methods 10](#_Toc484524122)

[Fig. 1: Overview Studyprotocol 12](#_Toc484524123)

[Tab. 1: Overview number of participants 12](#_Toc484524124)

[6.1 Description of experimental examination 13](#_Toc484524125)

[7. Risks and side effects of study interventions 15](#_Toc484524126)

[8. Goal criteria 15](#_Toc484524127)

[9. Data protection 16](#_Toc484524128)

[9.1. Database 16](#_Toc484524129)

[9.1.1. Data collection 16](#_Toc484524130)

[9.1.2. Patient inofrmation 16](#_Toc484524131)

[9.1.3. Data management 16](#_Toc484524132)

[9.2. Data coding and professional discretion 16](#_Toc484524133)

[10. Commuting accident insurance 17](#_Toc484524134)

[11. Information text and informed consent for participants 17](#_Toc484524135)

[12. References 17](#_Toc484524136)

[13. Appendix 19](#_Toc484524137)

# 1. List of abbreviations used in the test protocol

5-HT: 5-Hydroxytryptamin (serotonin)

BF: [breath](http://de.wikipedia.org/wiki/Atemfrequenz) frequency

BCAA: Branch-Chain Amino Acids

BfR: Bundesinstitut für Risikobewertung (federal institute for risk assessment)

NemV: Nahrungsergänzungsmittelverordnung (nutritional supplement enactment)

RPE: Rating of Perceived Exertion (subjective perception of effort)

Trp: tryptophan

VCO_2_: carbon dioxide release

VE: volume of exhalation

VO_2_: oxygen uptake

VO_2_ Max: maximal oxygen uptake capacity

# 2. Background

# 2.1 State of the art

# 2.1.1 Nutritional supplements in competitive sports

The constant aim for performance enhancement is present daily especially in sports. When a certain training volume is reached, athletes pursue diet optimization to enable performance enhancement. For this reason, athletes often reach for nutritional supplements.

According to the nutritional supplement enactment (NemV), nutritional supplements are by law not food, because they merely serve to complement the normal diet and not to replace it. Nutritional supplements are usually not used for energy, but rather are given in the form of capsules, pills or fizzy tablets and are made up of concentrated nutrients or other substance with physiological effect (NemV §1.1, 1-3) ^1^.

# 2.1.2 Nutritional supplements vs. functional food

Functional food is food, which aims to enhance health by adding certain ingredients. In Germany, law does not define the term functional food. While it is stated, that illness associated testimonies are prohibited, general health enhancement statements are tolerated ^2^.

Functional foods are „novel foods” as stated in the enactment (EG) No. 258/97 of the European Parliament and Council from January 27^th^ 1997 concerning novel foods and novel food ingredients ^3^. As a basic principle, they are only permitted on the market, if they pass through an appropriate European approval procedure.

According to the German association for consumer protection, nutrition and agriculture, functional foods are supposed to positively influence specific bodily functions which surpass the effect of the regular nutritional purpose, however, are exclusively food products and not pills ^4^.

The German federal institute for risk assessment (BfR) describes functional foods as distinct from nutritional supplements, because they are sold in the form of regular food products. Interchangeable are the terms „designer foods“ and nutraceuticals“ ^5^.

The best-known foods of this novel kind are probiotic dairy products, which favorably impact the gut flora through specific lactobacilli. Interestingly, enriched food product which do not explicitly point out their additional benefit, are not considered functional foods.

# 2.1.3 Central and peripheral fatigue and the roll of BCAAs

In supplementation for sports performance, the branched chain amino acids valine, leucine and isoleucine are of interest due to their role in central fatigue.

Physiological fatigue can originate both peripherally and centrally. Peripheral fatigue is, among others, caused by empty glycogen or phosphocreatine stores, the accumulation of protons and a non-conduction of neuromuscular signals. Peripheral fatigue has been well researched for decades ^6^.

The exact mechanisms of central fatigue, however, are being investigated now. One predominant theory is the Trp-5-HT central fatigue hypothesis. This hypothesis postulates, that an increase of 5-HT (serotonin) in the synapse and the resulting increase of post synaptic activation is responsible for central fatigue. The concentration of free tryptophan (fTrp) (precursor of 5-HT) in the blood determined the resulting 5-HT concentration in the synapse. Trp competes with BCAAs at the blood brain barrier for passage into the brain ^7^.

During longer endurance activities, glycogen stores are depleted and muscles switches to using BCAAs as an energy source. This causes an increase of the fTrp to BCAA ratio in the plasma. Thus, fTrp can more easily pass the blood brain barrier, as the competition for transporters is lower. Based on this theoretical construct it is assumed, that the ingestion of BCAA before and/or during activity enables a constant fTrp to BCAA ratio and therefore delays the rise of 5-HT in the synapse and the conjoined emergence of central fatigue ^8^.

# ****2.1.3 Food and the placebo effect****

Not only athletes attempt to optimize motor and cognitive performance capacity, body composition and wellbeing by ingesting certain foods or nutritional supplements. The degree to which expectations and knowledge about certain foods alone can influence performance capacity and subjective perception has only been marginally researched.

The few studies available in this research area have only looked at the placebo effect of isolated substances like glucose, amino acids, caffeine, alcohol and ginseng ^9–14^. None of these studies has investigated the effect of the interplay of all senses, which are influenced by the salience of the visual, gustatory, olfactory, tactile and auditory characteristics of a complete food products.

Harris and Johns investigated the placebo effect of the diet and nutrition culture in a review article ^15^. They analyzed the influence of taste, color, name and labeling of food products on emotions, expectations, associations and conditioned reactions. The authors’ conclusions based on more than 100 studies was, that the total effect of a food product, a meal or a diet rarely, if ever, is the result of the nutrient content or the chemically traceable bottom-up effect, but rather an interplay of all factors.

# 2.2 Study purpose

This study aims to investigate, whether a) there is an acute effect of BCAAs on endurance performance and b) whether the form of administration influences the degree of the performance enhancing effect of BCAAs. BCAAs will be administered in form of a nutritional supplement or in form of a functional food. Further, we are interested in how the athlete’s expectation regarding a specific nutritional supplement adds to changes in performance. Several psychometric questionnaires will also be measured.

# 3. Study goals

This study serves as a pilot study to investigate, if BCAAs have an acute effect on endurance performance and whether the form of administration of a supplement influences the degree of performance enhancement. The following hypotheses will be addressed:

**Hypothesis I (H_1_):** *BCAAs have an acute enhancing effect on endurance performance of athletes, which is measurable in subjective effort perception (rating of perceived exertion), objective effort and physiological effort.*

**Hypothesis II (H_2_):** *The form of administration of the supplement influences the degree of performance enhancement. It is expected, that the administration as a functional food will cause greater performance enhancement by way of visual, gustatory, olfactory and tactile salience compared to the administration of a nutritional supplement (capsule).*

# 4. Duration

Data collection is estimated to last from February to April 2014. For the experiment, we will recruit 30 participants; they will be tested on 3 days at the same time of day (morning, afternoon, evening). The entire test will take 6h total, 1h for the pretest and 2,5h each for test session 1 and 2. For total data collection time we estimate 180h (30 participants, 6h each).

# 5. Study population

# 5.1 Description of study population

Thirty competitive cycling, running or triathlon athletes will be tested.

# 5.2 Inclusion and exclusion criteria

Participants will be competitive cycling, running or triathlon athletes between 18 and 40 years of age, completing 3-5 training sessions per week and regularly participating in competitions. Ideally, the participants will be in the preparation phase for the competition season of their discipline.

The following exclusion criteria were set for the study: training pause for more than 4 weeks prior to testing, pregnant and breastfeeding women, regular consumption of pharmaceuticals, chronic and acute illness of the gastrointestinal tract, the central nervous system, the cardio pulmonary system or the otolaryngol system.

# 5.3 Recruiting

Potential participants will be recruited via bulletins and existing contacts through the walk-in clinic of the Department of Sports Medicine. Subjects are recruited on a voluntary basis. For study participation, they receive an expense allowance as well as the results of their performance tests.

# 5.4 Number of participants

Participant numbers in the research area of performance enhancing effects of supplements are usually between 5 and 10 subjects for each experimental group and these studies were able to show significant differences between supplement and placebo groups ^16–18^. As this is a pilot study, we expect that subject numbers of N=6 in each experimental group will provide clear results.

# 6. Study protocol and experimental methods

This is a randomized, double blind placebo controlled study to investigate the acute effect of BCAAs on endurance performance in athletes, administered in different forms. Participants are informed, that this is a randomized double-blind study, where neither the participant nor the experimenter knows, whether BCAAs or an inactive placebo is given. All testing will be carried out in the research laboratory of the Department of Sports Medicine at the University Hospital Tuebingen (Director: Prof. Dr. med. Andreas Nieß).

At the pretest, all participants receive an educational pamphlet and are informed about the study protocol. After reading the information and clearing up any answers, participants must sign the informed consent form to be permitted to participate in the study. At this point, participants are instructed to abstain from eating in the three hours immediately prior to the two test sessions and only drink water.

After signing the informed consent form, participants are randomized into 5 experimental groups (1: BCAA food, 2: placebo food, 3: BCAA supplement, 4: placebo supplement). At the first test session, participants perform an isokinetic time trial on the ergometer at 80% VO2 max for maximal duration. Ventilatory exchange, oxygen uptake, carbon dioxide exhalation, breath frequency and blood lactate concentration are measured.

At the second (intervention) test session, participants receive either BCAAs or placebo or no intervention before the same time trial. In the time between receiving the intervention and the start of the time trial, participants receive and information pamphlet about BCAAs to standardize expectations and knowledge among athletes. They have 10 minutes to read the pamphlet. Participants then fill out a short questionnaire, which measures the subjective expectations regarding the effect of BCAAs on their endurance performance.

The groups BCAA food and placebo food receive the intervention in form of a food product (pudding). The groups BCAA supplement and placebo supplement receive the intervention in form of a nutritional supplement (capsules). The control group receives no intervention. The only differences between food and supplement are mouth feel, gustation, olfaction and visual appearance. In terms of BCAA concentration both interventions are equal.

We expect an increase in performance at the second test session independent of intervention. This expected increase in performance is comprised of the natural effect of training and the high motivation regarding individual performance enhancement in a test setting among athletes. These factors, however, will influence all athletes to the same degree. Additionally, the control group controls for these confounding effects.

# Fig. 1: Overview study protocol

**
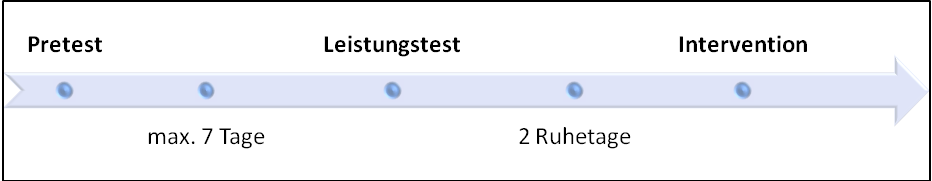
**

# Tab. 1: Overview number of participants

|  | **BCAAs** | **Placebo** | **Control** |
| --- | --- | --- | --- |
| **Food** | 6 | 6 | 6 |
| **Supplement** | 6 | 6 |  |

The intervention of either BCAAs or placebo are either given in the form of capsules or as a food product. These preparations are composed as follows:

Pudding: corn starch, vanilla flavor, stevia

BCAA: Valine, Isoleucine, Leucine as a ratio of 2:2:1

BCAA food (pudding with BCAA): total amount: 130g made up of 125g of pudding and 5g of BCAAs. In addition: 120ml water.

Placebo food (pudding without BCAA): total amount: 130g, made up of 130g of pudding. In addition: 120ml water.

BCAA supplement (capsules with BCAA): 4 capsules with 1,25g BCAAs each. In addition: 250ml water.

Placebo supplement (capsules without BCAA): 4 capsules with 1,25g corn starch each. In addition: 250ml water.

# 6.1 Description of experimental examinations

Performance diagnostics are performed using a spiro ergometer. Heart rate, breath and physiology are measure at rest and during activity. To measure performance at test session 1 and 2, participants are instructed to cycle as long as possible at an intensity of 80% of their VO_2_ max (maximal oxygen uptake). The individual VO_2_ max will be determined during the pretest using a ramping protocol.

During the spiro ergometric testing, participants wear a face mask, which measures the concentration of oxygen and carbon dioxide in the exhaled air. Attached to the breath mask is a volume sensor, which measure the total volume of inhaled and exhaled air. Part of the breath air goes through a tube to the gas sensor of the spirometry device, where the gas composition is analyzed and compared to the surrounding air. Thus, the ventilatory exchange (VE), volume of inhaled oxygen (VO_2_), volume of exhaled carbon dioxide (VCO2) and the breath frequency (BF) are measured. The maximal oxygen uptake (VO_2_ max) can be calculated using these measured values.

At the pretest, a ramping protocol is used to determine the VO_2_ max. In the process, the intensity (resistance) is steadily increased while the above-mentioned parameters are measured. In 3-minute intervals the blood lactate concentration is measured using a prick test at the ear lobe. The intensity is increased until the participant can no longer fulfill the performance expectations. The ramping protocol usually lasts 8-12 minutes.

The maximal temporal difference between the pretest and the first test session is 7 days, to ensure that the VO_2_ max is still valid and hasn’t significantly changed through training.

In addition, participants fill in the following questionnaires:

- SOQ: Sport Orientiation Questionnaire (SOQ) by Gill und Deeter (1988)
- AMS-Sport (Elbe, Wenhold & Müller, 2005): questionnaire for performance motivation in sports
- HOSP (Beckmann, 2003): questionnaire for task orientation in sports
- Questionnaire using a VAS measuring expectations of, experience with and prior knowledge of the supplement. Also: demographics and training details.

# 7. Risks and side effects of study interventions

Side effects from the ingestion of BCAAs are not expected. The dosage used in this study is harmless. Only in the case of severe over dosage, side effects such as nausea and gastro intestinal symptoms like diarrhea may occur. The spiro ergometric testing also bears no risks other than a possible reddening of the skin where the ECG electrodes were attached.

# 8. Goal criteria

The primary goal criteria of the study are the subjective (RPE) and the objective (duration and physiological parameters) performance. Test session 1 serves as a baseline for the dependent variables.

This is a 2 (food vs. supplement) x 2 (BCAAs vs. placebo) randomized factorial design with a no treatment control group. Differences in goal criteria between groups are analyzed using ANOVAs.

# 9. Data protection

# 9.1. Database

# 9.1.1. Data collection

Physiological endurance and psychometric data are collected. All data from the study are made anonymous and are then processed further. Data is only published in anonymous form.

# 9.1.2. Patient information

After finishing the study, participants are informed about their performance.

# 9.1.3. Data management

Data will be collected, analyzed and stored at the Department of Sports Medicine at the University Hospital Tuebingen. If a cooperation with other institutes will be necessary, the data will be passed on in anonymous form. The data will be stored for 10 years.

# 9.2. Data coding and professional discretion

Each participants record will be given a 6-digit code. A list with the code assignments and personal data will be kept in a separate cabinet, which is only accessible for the study leaders. For publications and follow up proposals all data will be in anonymous form. Everyone involved in the project must adhere to medical confidentiality.

# 10. Commuting accident insurance

A commuting accident insurance will be taken out.

# 11. Information text and informed consent for participants

See appendix.

# 12. References

1. Justiz, B. für. *Verordnung über Nahrungsergänzungsmittel (Nahrungsergänzungsmittel-verordnung-NemV)*. 1–7 (2011). at <http://books.google.com/books?hl=en&lr=&id=SKOG3V3PamoC&oi=fnd&pg=PA3&dq=Verordnung+%C3%BCber+Nahrungserg%C3%A4nzungsmittel+(+Nahrungserg%C3%A4nzungsmittelverordnung+-+NemV+)&ots=3Llu1-ufaC&sig=XwqcH33IqE5R_De454FNtwhSVS4>

2. Viell, B. Funktionelle Lebensmittel und Nahrungsergänzungsmittel. *Bundesgesundheitsblatt - Gesundheitsforsch. - Gesundheitsschutz* **44,** 193–204 (2001).

3. *Neuartige Lebensmittel und neuartige Lebensmittelzutaten*. *Amtsblatt Nr. …* (EG) Nr. 258/97 (Europäisches Parlament und Rat). at <http://www.biotechnologie.de/BIO/Redaktion/PDF/de/Richtlinien/b10__novel-food-verordnung,property=pdf,bereich=bio,sprache=de,rwb=true.pdf>

4. Funktionelle Lebensmittel (CD). *aid Infod. Ernährung, Landwirtschaft, Verbraucherschutz e. V.* BestellNr. 3816 at <http://www.aid.de/downloads/3816_funktionelle_lebensmittel_definition.pdf>

5. Gesundheitliche Bewertung funktioneller Lebensmittel. *Bundesinstitut für Risikobewertung* at <http://www.bfr.bund.de/de/gesundheitliche_bewertung_funktioneller_lebensmittel-152.html>

6. Finsterer, J. Biomarkers of peripheral muscle fatigue during exercise. *BMC Musculoskelet. Disord.* **13,** 218 (2012).

7. Newsholme, E. A. & Blomstrand, E. Branched-Chain Amino Acids and Central Fatigue. *J. Nutr.* 274S – 276S (2006).

8. Blomstrand, E. Amino acids and central fatigue. *Amino Acids* **20,** 25–34 (2001).

9. Beedie, C. J. & Foad, A. J. The placebo effect in sports performance: a brief review. *Sport. Med.* **39,** 313–29 (2009).

10. Mikalsen, a, Bertelsen, B. & Flaten, M. a. Effects of caffeine, caffeine-associated stimuli, and caffeine-related information on physiological and psychological arousal. *Psychopharmacology (Berl).* **157,** 373–80 (2001).

11. Green, M. W., Taylor, M. a., Elliman, N. a. & Rhodes, O. Placebo expectancy effects in the relationship between glucose and cognition. *Br. J. Nutr.* **86,** 173–179 (2001).

12. Chambers, E. S., Bridge, M. W. & Jones, D. a. Carbohydrate sensing in the human mouth: effects on exercise performance and brain activity. *J. Physiol.* **587,** 1779–94 (2009).

13. Sievenpiper, J. L., Ezatagha, A., Dascalu, A. & Vuksan, V. When a placebo is not a “placebo”: a placebo effect on postprandial glycaemia. *Br. J. Clin. Pharmacol.* **64,** 546–9 (2007).

14. Testa, M., Fillmore, M. & Norris, J. Understanding alcohol expectancy effects: Revisiting the placebo condition. *Alcohol. Clin. Exp. Res.* **30,** 339–348 (2006).

15. Harris, C. S. & Johns, T. The Total Food Effect : Exploring Placebo Analogies in Diet and Food Culture. *J. Mind Body Regul.* **1,** 143–160 (2011).

16. Peltier, S. L. *et al.* Effects of carbohydrates-BCAAs-caffeine ingestion on performance and neuromuscular function during a 2-h treadmill run: a randomized, double-blind, cross-over placebo-controlled study. *J. Int. Soc. Sports Nutr.* **8,** 22 (2011).

17. Duncan, M. J. THE EFFECT OF CAFFEINE INGESTION ON ANAEROBIC PERFORMANCE IN MODERATELY TRAINED ADULTS. **3,** 129–134 (2009).

18. Blomstrand, E., Hassmén, P., Ek, S., Ekblom, B. & Newsholme, E. a. Influence of ingesting a solution of branched-chain amino acids on perceived exertion during exercise. *Acta Physiol. Scand.* **159,** 41–9 (1997).
