# Supplementary material for: Increasing effort without noticing: A randomized controlled pilot study about the ergogenic placebo effect in endurance athletes and the role of supplement salience
Source: PLoS One. 2018 Jun 11;13(6):e0198388. doi: 10.1371/journal.pone.0198388 (PMC5995445; doi:10.1371/journal.pone.0198388)
Supplement: S3 File — (DOCX) [file pone.0198388.s003.docx]

Version 2

**„Akutwirkung eines Nahrungsergänzungsmittels auf die Ausdauerleistung - eine placebokontrollierte Doppelblindstudie“**

Studienleiter:

Prof. Dr. med. A. Nieß

Ärztlicher Direktor - Abteilung Sportmedizin

Medizinische Klinik - Universitätsklinikum Tübingen

Hoppe-Seyler-Str. 6

72076 Tübingen

Tel.: [07071-29 86493](tel:07071-29%2086493)

Fax: [07071-29 25028](tel:07071-29%2025028)

e-mail: [andreas.niess@med.uni-tuebingen.de](mailto:andreas.niess@med.uni-tuebingen.de)

<http://www.med.uni-tuebingen.de/sportmedizin/index.htm>

Weitere beteiligte Wissenschaftler:

Ellen Kristina Brölz, MSc

Finanzierung der Studie

Die Studie wird aus Mitteln der Abteilung finanziert. Drittmittel werden bei der Danone Stiftung (Sachmittel) sowie der Else-Kröner Fresenius Stiftung (Personalmittel) beantragt.

Tübingen, 10.01.2014

____________________________

Prof. Dr. med. A. Nieß

**Zusammenfassung**

Die Leistungssteigerung ist nicht nur das Ziel von professionellen Sportlern, sondern auch von Leistungssportlern und ambitionierten Hobbysportlern. Wenn das Training selbst bereits optimiert ist, werden oft andere Optionen gesucht die Leistung weiter zu steigern. Viele Sportler wollen aus diesem Grund auch Ihre Ernährung optimieren und greifen daher oft zu spezifischen Nahrungs- und Nahrungsergänzungsmitteln, von denen sie wissen bzw. glauben, dass sie eine bestimmte Auswirkung auf die Leistung, die Regeneration oder generell das Befinden haben.

In diesem Zusammenhang spielen Aminosäuren (AS), besonders die verzweigkettigen AS, eine große Rolle. Ihre unterstützenden Funktionen in der Regeneration sind gut untersucht jedoch fehlt es an Studien, die deren akute leistungssteigernde Wirkung untersuchen. Diese Studie soll diese Lücke schließen.

Es handelt sich um einen randomisierten placebokontrollierten doppelblinden Versuch zur Untersuchung der Akutwirkung eines Branch-Chain Amino Acid (verzweigkettige Aminosäuren) Präparats (BCAA-Präparat) auf die Ausdauerleistung bei gesunden Ausdauersportlern aus den Bereichen Rad-, Laufsport und Triathlon. Im Versuch werden das BCAA-Präparat und das Placebo-Präparat entweder in Kapselform (Supplement) oder in Pudding eingerührt (Food) verabreicht. Jeder Proband wird drei Mal getestet. Bei einem Pretest wird die individuelle maximale Sauerstoffaufnahmefähigkeit ermittelt. Am 1. Messzeitpunkt wird die Ausdauerleistung ohne Intervention und am 2. Messzeitpunkt nach vorheriger Verabreichung des BCAA- oder Placebo-Präparates getestet. Zwischen den beiden Messzeitpunkten liegen 2 Tage.

Die Probanden erhalten die Information, dass sie an einem randomisierten Doppelblindversuch teilnehmen, bei dem sie entweder ein Nahrungsergänzungsmittel oder ein Placebo bekommen. Erhoben werden neben den primären Endpunkten (subjektive und objektive Ausdauerleistung) auch die Erwartungen, die mit der Einnahme des BCAA-Präparates einhergehen. Vor und nach den spiroergometrischen Leistungstests erhalten alle Probanden eine psychometrische Testbatterie.

**Inhaltsverzeichnis**

[1. Liste der im Prüfplan verwendeten Abkürzungen 4](#_Toc376874898)

[2. Grundlagen 4](#_Toc376874899)

[2.1 Stand der Wissenschaft 4](#_Toc376874900)

[2.1.1 Nahrungsergänzungsmittel im Leistungssport 4](#_Toc376874901)

[2.1.2 Nahrungsergänzungsmittel vs. Funktionelle Nahrungsmittel 4](#_Toc376874902)

[2.1.3 Zentrale und periphere Ermüdung und die Rolle von BCAAs 5](#_Toc376874903)

[2.1.3 Nahrungsmittel und Placebowirkung 6](#_Toc376874904)

[2.2 Zusammenfassende Beschreibung und Begründung der Studie 6](#_Toc376874905)

[3. Ziele der Studie 6](#_Toc376874906)

[4. Studiendauer 7](#_Toc376874907)

[5. Studienpopulation 7](#_Toc376874908)

[5.1 Beschreibung der Studienpopulation 7](#_Toc376874909)

[5.2 Ein- und Ausschlusskriterien 7](#_Toc376874910)

[5.3 Rekrutierung 7](#_Toc376874911)

[5.4 Fallzahl 7](#_Toc376874912)

[6. Studienablauf und Untersuchungsmethoden 8](#_Toc376874913)

[Abb. 1: Übersicht Studienablauf 9](#_Toc376874914)

[Tab. 1: Übersicht Probandenzahl 9](#_Toc376874915)

[6.1 Aufstellung studienbedingter Untersuchungen 9](#_Toc376874916)

[7. Risiken und Nebenwirkungen studienbedingter Eingriffe 10](#_Toc376874917)

[8. Zielkriterien 10](#_Toc376874918)

[9. Datenschutz 11](#_Toc376874919)

[9.1. Datenbank 11](#_Toc376874920)

[9.1.1. Datenerfassung 11](#_Toc376874921)

[9.1.2. Patienteninformation 11](#_Toc376874922)

[9.1.3. Datenverwaltung 11](#_Toc376874923)

[9.2. Datenverschlüsselung und Wahrung der ärztlichen Schweigepflicht 11](#_Toc376874924)

[10. Wegeunfallversicherung 11](#_Toc376874925)

[11. Aufklärungstext und Einverständniserklärung für Studienteilnehmer 11](#_Toc376874926)

[12. Literaturverzeichnis 11](#_Toc376874927)

[13. Anhang 13](#_Toc376874928)

# 1. Liste der im Prüfplan verwendeten Abkürzungen

5-HT: 5-Hydroxytryptamin (Serotonin)

AF: [Atemfrequenz](http://de.wikipedia.org/wiki/Atemfrequenz)

BCAA: Branch-Chain Amino Acids (Verzweigkettige Aminosäuren)

BfR: Bundesinstitut für Risikobewertung

NemV: Nahrungsergänzungsmittelverordnung

RPE: Rating of Perceived Exertion (subjektive Anstrengungseinschätzung)

Trp: Tryptophan

VCO_2_: Kohlendioxidabgabe

VE: [Atemminutenvolumen](http://de.wikipedia.org/wiki/Atemminutenvolumen)

VO_2_: Sauerstoffaufnahme

VO_2_ Max: maximale Sauerstoffaufnahmefähigkeit

# 2. Grundlagen

# 2.1 Stand der Wissenschaft

# 2.1.1 Nahrungsergänzungsmittel im Leistungssport

Gerade im Sport ist das konstante Streben nach Leistungssteigerung tagtäglich präsent. Ab einem gewissen Trainingspensum wird meist zusätzlich zum Training eine Ernährungsoptimierung angestrebt, um eine Leistungssteigerung zu ermöglichen. Aus diesem Grund greifen Sportler oft zu Nahrungsergänzungsmitteln.

Nahrungsergänzungsmittel sind laut der Nahrungsergänzungsmittelverordnung (NemV) rein rechtlich gesehen Lebensmittel, denn sie dienen lediglich der Ergänzung der normalen Ernährung und nicht deren Ersatz. Nahrungsergänzungsmittel werden nicht in erster Linie zur Energieversorgung herangezogen, sondern werden in Form von Kapseln, Pastillen, Tabletten, Pillen oder Brausetabletten eingenommen und bestehen entweder aus konzentrierten Nährstoffen oder anderen Substanzen mit physiologischer Wirkung (NemV §1.1, 1-3)^1^.

# 2.1.2 Nahrungsergänzungsmittel vs. Funktionelle Nahrungsmittel

Als funktionelle Lebensmittel bezeichnet man solche Nahrungsmittel, die durch die Zugabe von bestimmten Inhaltsstoffen gesundheitsfördernde Effekte erzielen sollen. In Deutschland sind funktionelle Lebensmittel nicht rechtlich definiert. Es ist zwar festgelegt, dass keine krankheitsbezogenen Aussagen gemacht werden dürfen, doch werden allgemeine gesundheitsfördernde Aussagen geduldet^2^.

Funktionelle Lebensmittel entsprechen „neuartigen Lebensmitteln“ im Sinne der Verordnung (EG) Nr. 258/97 des Europäischen Parlaments und des Rates vom 27. Januar 1997 über neuartige Lebensmittel und neuartige Lebensmittelzutaten^3^. Sie dürfen grundsätzlich nur auf den Markt kommen, wenn sie ein entsprechendes europäisches Genehmigungsverfahren durchlaufen haben.

Laut des Vereins für Verbraucherschutz, Ernährung, Landwirtschaft e.V. sollen funktionelle Lebensmittel bestimmte Körperfunktionen über den reinen Ernährungszweck hinaus günstig beeinflussen und sollen bei den üblichen Verzehrmengen wirken, sind jedoch ausschließlich Lebensmittel und keine Pillen^4^.

Das Bundesinstitut für Risikobewertung (BfR) beschreibt funktionelle Lebensmittel als distinkt von Nahrungsergänzungsmitteln, da sie in den typischen Nahrungsmittelformen verkauft werden. Synonyme sind "Designer-Lebensmittel" und "Nutraceuticals"^5^.

Die bekanntesten Lebensmitte dieser neuen Art sind probiotische Milchprodukte, die durch bestimmte Arten von Milchsäurebakterien die Darmflora günstig beeinflussen sollen. Interessanterweise gelten angereicherte Lebensmittel die keinen expliziten Hinweis auf ihren Zusatznutzen aufzeigen nicht als funktionelle Lebensmittel.

# 2.1.3 Zentrale und periphere Ermüdung und die Rolle von BCAAs

Interessant im Bereich der Supplementierung bei Sportlern sind die verzweigkettigen Aminosäuren (Branch-Chain Amino Acids (BCAAs)) Valin, Leucin und Isoleucin und ihre Rolle in zentralen Ermüdungszuständen (central fatigue).

Physiologische Ermüdung kann peripheren und zentralen Ursprungs sein. Erstere ist unter anderem auf leere Glykogen- oder Phosphocreatinspeicher, der Ansammlung von Protonen und einer Nichtübertragung neuromuskulärer Signale zurückzuführen. Die periphere Ermüdung ist seit Jahrzehnten gut erforscht^6^.

Die genauen Mechanismen der zentralen Ermüdung hingegen werden derzeit erforscht. Die vorherrschende Theorie ist die Trp-5HT central-fatigue – Hypothese. Diese Hypothese postuliert, dass der Anstieg von 5-HT (Serotonin) in der Synapse und die daraus resultierende zunehmende Aktivierung des postsynaptischen Neurons für zentrale Ermüdungserscheinungen verantwortlich sind. Die Konzentration von freiem Tryptophan (Trp) (Vorläufer von 5-HT) im Blut bestimmt die daraus resultierende 5-HT Konzentration in den Synapsen. Trp konkurriert an der Blut-Hirn-Schranke mit den Verzweigkettigen Aminosäuren Valin, Leucin und Isoleucin (BCAAs) um die Passage ins Gehirn^7^.

Während längerer Ausdauerbelastung leeren sich die Glykogenspeicher und die Muskulatur steigt zunehmend auf BCAAs als Energielieferant um. Dadurch steigt das Verhältnis von freiem Trp (fTrp) zu BCAAs im Plasma an. Dadurch kann fTrp die Blut-Hirn-Schranke leichter passieren, da die Konkurrenz um die Transporter geringer wird. Aus dieser theoretischen Grundlage lässt sich schließen, dass die Einnahme von BCAAs vor und/ oder während der Belastung dazu führen, dass das Verhältnis von fTrp zu BCAAs konstant bleibt und so den Anstieg von 5-HT in den Synapsen und die damit verbundenen zentralen Erschöpfungserscheinungen verzögert^8^.

# ****2.1.3 Nahrungsmittel und Placebowirkung****

Nicht nur Athleten versuchen ihre motorische und kognitive Leistungsfähigkeit, sowie ihre Körperkomposition und ihr Wohlbefinden durch die Einnahme von bestimmten Nahrungs- oder Nahrungsergänzungsmitteln zu optimieren. Inwiefern die Erwartungen und das Wissen über bestimmte Nahrungsmittel alleine die Leistungsfähigkeit und die subjektive Wahrnehmung beeinflussen können ist bislang jedoch nur marginal erforscht.

Die wenigen Studien, die es in diesem Bereich gibt haben nur die Placebowirkung isolierter Substanzen, wie zum Beispiel Glukose, Aminosäuren, Koffein, Alkohol und Ginseng untersucht^9–14^. Keine dieser Studien hat jedoch untersucht, wie sich das Zusammenspiel aller Sinne auswirkt, welche durch die Salienz der visuellen, gustatorischen, olfaktorischen, taktilen und auch auditiven Reize eines vollständigen Nahrungsmittels beeinflusst werden.

Harris und Johns untersuchten in einer Übersichtsarbeit das Thema der Placebowirkung in der Diät- und Ernährungskultur^15^. Sie analysierten den Einfluss von Geschmack, Farbe, Name und Beschriftung von Lebensmitteln auf Emotionen, Erwartungen, Assoziationen und konditionierte Reaktionen. Das Fazit der Autoren aus den über 100 erwähnten Studien ist, dass der totale Effekt eines Nahrungsmittels, einer Mahlzeit oder einer Diät nur selten, wenn je, das reine Ergebnis des Nährstoffgehalts oder der chemisch nachvollziehbaren bottom-up Wirkung ist, sondern ein Zusammenspiel der Faktoren ist.

# 2.2 Zusammenfassende Beschreibung und Begründung der Studie

Diese Studie soll erfassen, ob a) eine Akutwirkung von BCAAs im Ausdauerbereich erkennbar ist und b) ob die Darreichungsform einen Einfluss auf das Ausmaß der leistungssteigernden Akutwirkung von BCAAs hat. Die BCAAs sollen einmal als Nahrungsergänzungsmittel (Supplement) und einmal in Form eines funktionellen Lebensmittels (Food) gegeben werden. Dabei interessiert unter anderem, wie die Erwartungen der Sportler selbst über bestimmte Nahrungsergänzungsmittel die Leistung zusätzlich beeinflussen können. Weiterhin wird eine Fragebogen-Batterie zu psychometrischen Maßen erhoben.

# 3. Ziele der Studie

Diese Studie dient als Pilotstudie, um zu erfassen, ob BCAAs eine Akutwirkung im Ausdauerbereich haben und ob die Darreichungsform eines Supplements einen Einfluss auf das Ausmaß der Leistungssteigerung hat. Folgende Hypothesen sollen adressiert werden:

**Hypothese I (H_1_):** *BCAAs haben eine steigernde Akutwirkung auf die Ausdauerleistung von Sportlern, welche sich in der Belastungsdauer bei 80% des individuellen VO2 Max, in der subjektiven Anstrengungseinschätzung - Rating of Perceived Exertion (RPE) und in den physiologischen Parametern widerspiegelt.*

**Hypothese II (H_1_):** *Die Darreichungsform des Supplements hat einen Einfluss auf das Ausmaß der Leistungssteigerung. Hierbei wird erwartet, dass die Darreichung in Form eines Nahrungsmittels durch die Salienz seiner visuellen, gustatorischen, olfaktorischen und taktilen Merkmale eine größere Leistungssteigerung bewirkt als in Form eines Supplements (Kapsel).*

# 4. Studiendauer

Die Untersuchungen werden sich voraussichtlich von Februar bis April 2014 erstrecken. Für den Versuch werden 30 Versuchspersonen rekrutiert; sie werden an je 3 Tagen zur gleichen Tageszeit (morgens, nachmittags oder abends) untersucht. Die Messung dauert pro Proband insgesamt ca. 6 h, davon 1h für den Pretest, 2 ½ h an Messtag 1 und 2 ½ h an Messtag 2. Die reine Messzeit der Gesamtstudie wird somit auf ca. 180 h (30 Probanden à 6 Stunden) angesetzt.

# 5. Studienpopulation

# 5.1 Beschreibung der Studienpopulation

Es werden 30 Leistungssportler aus Rad- und Laufsport und gegebenenfalls Triathlon untersucht.

# 5.2 Ein- und Ausschlusskriterien

Probanden sollen zwischen 18 und 40 Jahre alt sein, beiderlei Geschlechts und ambitionierte Sportler aus Rad- und Laufsport und gegebenenfalls Triathlon sein (Training 3-5 Mal pro Woche, regelmäßige Wettkampfteilnahme). Idealerweise sollten sich die Probanden in der Saisonvorbereitung für Ihre jeweilige Disziplin befinden.

Folgende Kriterien führen zum Ausschluss aus der Studie: Trainingspause länger als 4 Wochen vor Testbeginn, schwangere und stillende Frauen, akute Medikamenteneinnahme, chronische und akute Erkrankungen des Verdauungstraktes, des ZNS, des kardio-pulmonalen System und des Hals-Nasen-Ohren-Bereiches.

# 5.3 Rekrutierung

Die potentiellen Probanden sollen durch Aushänge (siehe Anhang) und/oder bereits bestehenden Kontakt über die sportmedizinische Ambulanz rekrutiert werden. Die Rekrutierung der Personen basiert auf absoluter Freiwilligkeit. Für die Versuchsbeteiligung erhalten die Probanden jeweils eine Aufwandsentschädigung, sowie die Ergebnisse ihres Leistungstests.

# 5.4 Fallzahl

Fallzahlen im Bereich der leistungssteigernden Wirkung von Supplementen sind meistens im Bereich zwischen 5 und 10 Probanden pro Experimentalgruppe und konnten einen signifikanten Unterschied zwischen Supplement und Placebo zeigen^16–18^. Da es sich hier um eine Pilotstudie handelt, ist davon auszugehen dass Probandenzahlen von N=6 in den Experimentalgruppen klare Ergebnisse liefern werden.

# 6. Studienablauf und Untersuchungsmethoden

Es handelt sich um eine randomisierte, doppelblinde, placebokontrollierte Studie zur akuten Wirkung von BCAAs in unterschiedlicher Darreichungsform auf die Ausdauerleistung von Sportlern. Die Probanden erhalten die Information, dass es sich um eine randomisierte Doppelblindstudie handelt, bei dem weder der Proband, noch der Versuchsleiter weiß, ob das Supplement (BCAA-Präparat) oder ein wirkstofffreies Placebo verabreicht wird. Das Experiment wird im Forschungslabor der Abteilung für Sportmedizin, Universitätsklinikum Tübingen (Leitung: Prof. Dr. med. Andreas Nieß) durchgeführt.

Am Pretest erhalten alle Probanden ein Aufklärungsschreiben (siehe Anhang) und werden über den weiteren Verlauf der Studie informiert. Nachdem der Proband die Informationen gelesen hat und mögliche Fragen geklärt wurden muss er die Einverständniserklärung unterschreiben, um an der Studie teilnehmen zu dürfen. An dieser Stelle werden die Probanden instruiert an beiden folgenden Messtagen in den 3 Stunden vor dem Versuch keine Nahrung mehr zu sich zu nehmen und nur noch Wasser zu trinken.

Die Probanden werden nach schriftlichem Einverständnis nach dem Zufallsprinzip einer der 5 Versuchsgruppen (1: BCAA Food, 2: Placebo Food, 3: BCAA Supplement, 4: Placebo Supplement, 5: Kontrolle) zugewiesen. Am 1. Messtag wird ein Spiroergometer-Test auf dem Fahrrad durchgeführt, bei dem der Proband bei 80% seines beim Pretest bestimmten VO2 Max so lange wie möglich radeln muss. Gemessen werden das [Atemminutenvolumen](http://de.wikipedia.org/wiki/Atemminutenvolumen) (VE), die Sauerstoffaufnahme (VO2), die Kohlendioxidabgabe (VCO2) und die [Atemfrequenz](http://de.wikipedia.org/wiki/Atemfrequenz) (AF), sowie die Laktatkonzentration im Blut über einen Pricktest am Ohrläppchen. Wichtig ist auch die Dauer, die der Proband bei 80% seines VO_2_ Max durchgehalten hat.

Bei der Intervention (2. Messtag) erhalten die Probanden entweder BCAAs oder ein Placebo-Präparat 60 Minuten vor dem Fahrrad-Spiroergometer-Test. In der Zeit bis zum Versuch erhalten die Probanden eine Informationsbroschüre über BCAAs, um die Erwartungen und das Wissen zwischen den Probanden auf einen Stand zu bringen. Sie haben 10 Minuten Zeit die Broschüre anzuschauen. Danach werden die Probanden gebeten, eine psychometrische Testbatterie auszufüllen. Dies schließt auch die subjektive Erwartung der Auswirkungen der BCAAs auf die eigene Ausdauerleistung ein.

Die Gruppen BCAA Food und Placebo Food erhalten die Intervention in Form eines Nahrungsmittels (Pudding). Die Gruppen BCAA Supplement und Placebo Supplement erhalten die Intervention in Form eines Supplements (Kapsel). Die Kontrollgruppe erhält keine Intervention. Das Nahrungsmittel und das Supplement unterscheiden sich lediglich in ihrem Mundgefühl, Geschmack, Geruch und Aussehen. Ansonsten sind Kaloriengehalt und BCAA Konzentration (bei den Verum-Präparaten) beider Interventionen gleich.

Es ist davon auszugehen, dass sich am 2. Messtag eine Leistungssteigerung unabhängig von der Intervention abzeichnet. Diese zu erwartende Steigerung setzt sich zum einem aus dem natürlichen Trainingseffekt und zum anderen aus der hohen Motivation zur individuellen Leistungssteigerung insbesondere unter Testbedingungen bei Sportlern zusammen. Diese Faktoren wirken jedoch auf alle Probanden gleichermaßen. Zusätzlich sorgt die Kontrollgruppe dafür, dass für solche interventionsunabhängigen Effekte kontrolliert werden kann.

# Abb. 1: Übersicht Studienablauf

**
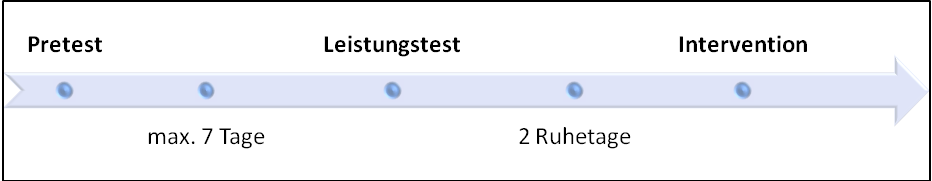
**

# Tab. 1: Übersicht Probandenzahl

|  | **BCAA-Präparat** | **Placebo** | **Kontrolle** |
| --- | --- | --- | --- |
| **Food** | 6 | 6 | 6 |
| **Supplement** | 6 | 6 |  |

Bei den Studienpräparaten handelt es sich um ein BCAA-Präparat bzw. ein Placebo-Präparat, das entweder in Kapselform oder in Lebensmittelform verabreicht wird. Die Zusammensetzung der Präparate sieht wie folgt aus:

Pudding: Maisstärke, natürlicher Vanillegeschmack, Stevia

BCAA: Valin, Isoleucin, Leucin im Verhältnis 2:2:1

BCAA Food (Pudding mit BCAA): Gesamtmenge: 130g, davon 125g Pudding und 5 g BCAAs. Dazu 120ml Wasser.

Placebo Food (Pudding ohne BCAA): Gesamtmenge: 130g, davon 130g Pudding. Dazu 120ml Wasser.

BCAA Supplement (Kapseln mit BCAA): 4 Kapseln à 1,25 g BCAA. Dazu 250ml Wasser.

Placebo Supplement (Kapseln ohne BCAA): 4 Kapseln à 1,25g Maisstärke. Dazu 250ml Wasser.

# 6.1 Aufstellung studienbedingter Untersuchungen

Die Leistungsdiagnostik wird mithilfe eines Fahrrad-Spiroergometers durchgeführt. Hierbei wird die Funktion von Herz, Kreislauf, Atmung und muskulärem Stoffwechsel in Ruhe und unter körperlicher Belastung aufgezeichnet. Zur Leistungserfassung an Messtag 1 und 2 sollen Probanden so lange wie möglich bei 80% ihres VO_2_-Max (maximale Sauerstoffaufnahmefähigkeit) auf dem Ergometer durchhalten. Der individuelle VO_2_-Max wird bei einem Pretest mittels eines Rampen-Protokolls ermittelt.

Bei der spiroergometrischen Messung trägt der Proband eine Atemmaske, über die die Konzentrationen von Sauerstoff und Kohlendioxid in der Atemluft gemessen wird. An die Atemmaske ist ein Volumensensor zur Messung des ein- und ausgeatmeten Luftvolumens angebracht. Über einen Schlauch wird ein Teil der Atemluft zu den Gassensoren im Spiroergometrie-Gerät geleitet, Dort wird die Gaszusammensetzung analysiert und mit der Umgebungsluft verglichen. Gemessen wird also das [Atemminutenvolumen](http://de.wikipedia.org/wiki/Atemminutenvolumen) (VE), die Sauerstoffaufnahme (VO_2_), die Kohlendioxidabgabe (VCO_2_) und die [Atemfrequenz](http://de.wikipedia.org/wiki/Atemfrequenz) (AF). Aus all diesen Messwerten lässt sich die maximale Sauerstoffaufnahmefähigkeit (VO_2_ Max) bestimmen.

Beim Pretest wird ein Rampenprotokoll durchgeführt, welches zur Ermittlung der individuellen maximalen Sauerstoffaufnahmefähigkeit eingesetzt wird. Dabei wird die Belastungsintensität stetig erhöht und die oben genannten Parameter werden gemessen. In 3 minütigen Intervallen wird mittels eines Pricktest am Ohrläppchen die Laktatkonzentration im Blut bestimmt. Der Belastung wird solange gesteigert bis der Proband der Leistungsanforderung nichtmehr gerecht werden kann. Ein Rampentest dauert durchschnittlich 8-12 Minuten.

Der zeitliche Abstand zwischen Pretest und Messzeitpunkt 1 darf maximal 7 Tage betragen, damit die VO_2_-Max Werte bei den Leistungstests valide sind und sich nicht durch das normale Training verändert haben.

Darüber hinaus werden folgende Fragebögen erhoben:

- SOQ (Elbe, 2001) Fragebogen zur Bestimmung der Sportlichen Leistungsorientierungen: Deutsche Übersetzung des Sport Orientiation Questionnaire (SOQ) von Gill und Deeter (1988)
- AMS-Sport (Elbe, Wenhold & Müller, 2005) Fragebogen zum Leistungsmotiv im Sport
- HOSP (Beckmann, 2003) Fragebogen zur Handlungsorientierung im Sport
- Selbsterstellte Fragebögen mit visueller Analogskala (VAS) bezüglich Erwartungen (pre) und Erfahrung (post), bisherigem Wissensstand bezüglich Supplementen, Demographie, Trainingsplan und Ernährung.

# 7. Risiken und Nebenwirkungen studienbedingter Eingriffe

Nebenwirkungen sind bei der Einnahme von verzweigkettigen Aminosäuren nicht zu erwarten. Die hier verwendete Dosierung ist gänzlich unbedenklich. Nur bei einer Überdosierung kann es zu Übelkeit und Gastrointestinalen Symptomen wie Durchfall kommen. Auch die spiroergometrische Untersuchung birgt keine Risiken, außer möglicherweise einer leichten Rötung der Haut an den Elektrodenklebestellen.

# 8. Zielkriterien

Primäres Zielkriterium der Untersuchung ist die subjektive (RPE, Fragebögen) und die objektive (Belastungsdauer und ergometrische Leistungsparameter) Leistung bzw. Leistungssteigerung. Messzeitpunkt 1 dient hierbei als Baseline für die abhängigen Variablen.

Es handelt sich bei dieser Studie um ein 2 (Food vs. Supplement) x 2 (Verum vs. Placebo) randomisiert faktorielles Design mit einer no-treatment Kontrollgruppe. Unterschiede in den Zielkriterien zwischen den Gruppen werden mittels einer zweifaktoriellen Varianzanalyse (ANOVA) berechnet.

# 9. Datenschutz

# 9.1. Datenbank

# 9.1.1. Datenerfassung

Es werden physiologische Ausdauerleistungsdaten erhoben, sowie einige psychometrische Daten. Alle Daten aus der Studie, soweit sie für die wissenschaftliche Auswertung notwendig sind, werden pseudonymisiert und in dieser Form weiter verarbeitet. Eine Veröffentlichung erfolgt nur in pseudonymisiert Form.

# 9.1.2. Patienteninformation

Die Probanden erhalten nach Studienabschluss Auskunft über ihre Leistung.

# 9.1.3. Datenverwaltung

Die Daten werden in der Abteilung für Sportmedizin des Universitätsklinikums Tübingen erfasst, ausgewertet und aufbewahrt. Sollte eine Kooperation mit anderen Instituten notwendig werden, so werden die Daten pseudonymisiert weitergegeben. Die Daten sollen für 10 Jahre gespeichert werden.

# 9.2. Datenverschlüsselung und Wahrung der ärztlichen Schweigepflicht

Jeder Probanden-Akte wird ein zufällig erzeugter 6stelliger Code zugeordnet. Eine Liste mit der Zuordnung von Code und persönlichen Daten wird getrennt in einem separaten Schrank aufbewahrt zu dem nur die Studienleiter Zugang haben. Nach außen (Veröffentlichung, Verwendung für einen Nachfolgeantrag etc.) werden alle Daten vollständig anonymisiert. Alle am Projekt beteiligten Mitarbeiter unterliegen der ärztlichen Schweigepflicht.

# 10. Wegeunfallversicherung

Eine Wegunfallversicherung wird abgeschlossen.

# 11. Aufklärungstext und Einverständniserklärung für Studienteilnehmer

Siehe Anhang

# 12. Literaturverzeichnis

1. Justiz, B. für. *Verordnung über Nahrungsergänzungsmittel (Nahrungsergänzungsmittel-verordnung-NemV)*. 1–7 (2011). at <http://books.google.com/books?hl=en&lr=&id=SKOG3V3PamoC&oi=fnd&pg=PA3&dq=Verordnung+%C3%BCber+Nahrungserg%C3%A4nzungsmittel+(+Nahrungserg%C3%A4nzungsmittelverordnung+-+NemV+)&ots=3Llu1-ufaC&sig=XwqcH33IqE5R_De454FNtwhSVS4>

2. Viell, B. Funktionelle Lebensmittel und Nahrungsergänzungsmittel. *Bundesgesundheitsblatt - Gesundheitsforsch. - Gesundheitsschutz* **44,** 193–204 (2001).

3. *Neuartige Lebensmittel und neuartige Lebensmittelzutaten*. *Amtsblatt Nr. …* (EG) Nr. 258/97 (Europäisches Parlament und Rat). at <http://www.biotechnologie.de/BIO/Redaktion/PDF/de/Richtlinien/b10__novel-food-verordnung,property=pdf,bereich=bio,sprache=de,rwb=true.pdf>

4. Funktionelle Lebensmittel (CD). *aid Infod. Ernährung, Landwirtschaft, Verbraucherschutz e. V.* BestellNr. 3816 at <http://www.aid.de/downloads/3816_funktionelle_lebensmittel_definition.pdf>

5. Gesundheitliche Bewertung funktioneller Lebensmittel. *Bundesinstitut für Risikobewertung* at <http://www.bfr.bund.de/de/gesundheitliche_bewertung_funktioneller_lebensmittel-152.html>

6. Finsterer, J. Biomarkers of peripheral muscle fatigue during exercise. *BMC Musculoskelet. Disord.* **13,** 218 (2012).

7. Newsholme, E. A. & Blomstrand, E. Branched-Chain Amino Acids and Central Fatigue. *J. Nutr.* 274S – 276S (2006).

8. Blomstrand, E. Amino acids and central fatigue. *Amino Acids* **20,** 25–34 (2001).

9. Beedie, C. J. & Foad, A. J. The placebo effect in sports performance: a brief review. *Sport. Med.* **39,** 313–29 (2009).

10. Mikalsen, a, Bertelsen, B. & Flaten, M. a. Effects of caffeine, caffeine-associated stimuli, and caffeine-related information on physiological and psychological arousal. *Psychopharmacology (Berl).* **157,** 373–80 (2001).

11. Green, M. W., Taylor, M. a., Elliman, N. a. & Rhodes, O. Placebo expectancy effects in the relationship between glucose and cognition. *Br. J. Nutr.* **86,** 173–179 (2001).

12. Chambers, E. S., Bridge, M. W. & Jones, D. a. Carbohydrate sensing in the human mouth: effects on exercise performance and brain activity. *J. Physiol.* **587,** 1779–94 (2009).

13. Sievenpiper, J. L., Ezatagha, A., Dascalu, A. & Vuksan, V. When a placebo is not a “placebo”: a placebo effect on postprandial glycaemia. *Br. J. Clin. Pharmacol.* **64,** 546–9 (2007).

14. Testa, M., Fillmore, M. & Norris, J. Understanding alcohol expectancy effects: Revisiting the placebo condition. *Alcohol. Clin. Exp. Res.* **30,** 339–348 (2006).

15. Harris, C. S. & Johns, T. The Total Food Effect : Exploring Placebo Analogies in Diet and Food Culture. *J. Mind Body Regul.* **1,** 143–160 (2011).

16. Peltier, S. L. *et al.* Effects of carbohydrates-BCAAs-caffeine ingestion on performance and neuromuscular function during a 2-h treadmill run: a randomized, double-blind, cross-over placebo-controlled study. *J. Int. Soc. Sports Nutr.* **8,** 22 (2011).

17. Duncan, M. J. THE EFFECT OF CAFFEINE INGESTION ON ANAEROBIC PERFORMANCE IN MODERATELY TRAINED ADULTS. **3,** 129–134 (2009).

18. Blomstrand, E., Hassmén, P., Ek, S., Ekblom, B. & Newsholme, E. a. Influence of ingesting a solution of branched-chain amino acids on perceived exertion during exercise. *Acta Physiol. Scand.* **159,** 41–9 (1997).
