# Supplementary material for: Increasing effort without noticing: A randomized controlled pilot study about the ergogenic placebo effect in endurance athletes and the role of supplement salience
Source: PLoS One. 2018 Jun 11;13(6):e0198388. doi: 10.1371/journal.pone.0198388 (PMC5995445; doi:10.1371/journal.pone.0198388)
Supplement: S4 File — (DOCX) [file pone.0198388.s004.docx]

Medizinische Fakultät

Medizinische Klinik V

Abteilung Sportmedizin

Univ.-Prof. Dr. med. A. Nieß

Ärztlicher Direktor

Lehrstuhl für Sportmedizin

Telefon +49 7071 29 - 86 493

Telefax +49 7071 29 - 25 028

andreas.niess@med.uni-tuebingen.de

Universitätsklinikum Tübingen · Medizinische Klinik V (Sportmedizin)

Hoppe-Seyler-Str. 6 **^.^** 72076 Tübingen

**Contact:**

Ellen K. Broelz

ellen.broelz@med.uni-tuebingen.de

Mobil: 0178 2178 010

**Information sheet for the study: „Acute effect of a nutritional supplement on endurance performance – a placebo controlled double blind study“**

Dear potential study participants,

we are happy about your interest in participating in our study „Acute effect of a nutritional supplement on endurance performance – a placebo controlled double blind study“. In this study we aim to show, that branched chain amino acids (BCAAs), which are well known for their positive influence on recovery, can acutely enhance endurance performance. The performance enhancing effect is based on both peripheral and central processes. Until now there are only few studies, which have researched the acute effect of these amino acids in the endurance domain. This study is conducted by Ms. E. Broelz from the Department of Sports Medicine at the University Hospital Tübingen and supervised by Prof. Dr. med. A. Nieß and Dipl.-Sportwiss. P. Schneeweiss.

Study participation

We are looking for healthy competitive male endurance athletes from the following disciplines: cycling and triathlon between the age of 18 and 40 years. You should be performance oriented, complete 3-5 cycling training sessions weekly and regularly participate in competitions.

At the time of the tests, there should be no serious illness or use of medication. Further, there should be no training pause in the past 4 weeks prior to testing. If this was the case, please inform us.

Study procedure

Study duration and location

The study will last from February to June 2014 and will be carried out in 4 test sessions within 2-4 weeks. All tests will take place at the ergometry laboratory of the Department of Sports Medicine Tuebingen. For this study, it is necessary that you show up fasted on day 3 and 4 of testing, which means no food for 12 h and no coffee or anything containing caffeine.


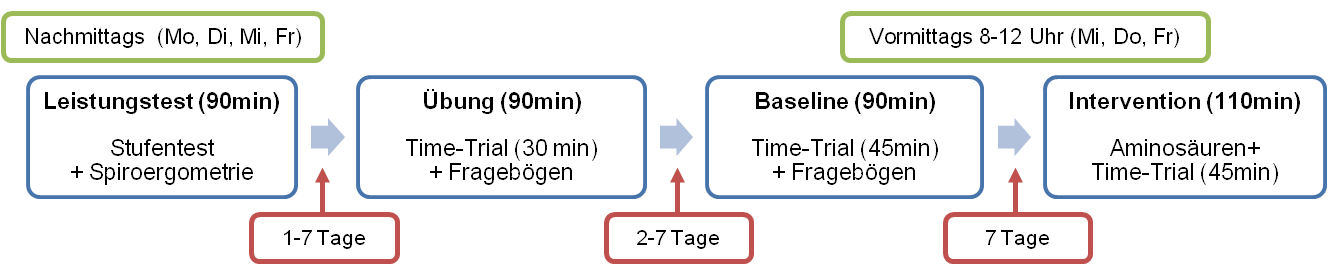
Overview

First, we ask you to read the information consent form and in case you decide to participate, please sign it. You will then be randomly assigned to either the BCAA, the placebo or the control group. Depending on the group you will receive either BCAAs, a placebo or no intervention on the 4^th^ day of testing.

General aspects

You should bring your regular cycling outfit including your cycling shoes on all test days. There are changing rooms and showers on site. **Important**: Please bring your own pedals (matching your shoes).

Performance test

T1: Duration: 90min [performance diagnostics]

AT the beginning of the study, we will measure your resting ECG, height and weight. Then you will be examined by the doctor, who will clear you for participation in the study. Afterwards, the performance diagnostics begin. You will cycle on an ergometer following a classical incremental performance test protocol. You can freely choose your seating position on the ergometer. Please find your preferred seating position before the protocol begins. The protocol runs as follows: You will start at a resistance of 40 Watt. The intensity will increase by 40 Watt every 3 minutes. You will keep going until exhaustion or other bodily ailments occur. You will freely determine when you want to stop the test. At the same time, we will measure ECG, lactate, and spirometry. For the spirometry, you will wear a respiratory mask to measure breath gasses, which will serve as an objectification of your cardio pulmonary performance. For the blood lactate diagnostics, we will take a blood sample (20µl) from your ear lobe at the end of every increment and at the end of the test.

T2: Duration 90min [test time trial (duration: 30min)]

On the second testing day, you will perform a 30min test time trial to get used to the isokinetic properties of the SRM cycling ergometer. You will cycle at a constant cadence of 95 revolutions per minute (rpm) with the goal of reaching your best possible performance. During the 10 min warm up and cool down you may choose your cadence freely between 70 and 100 rpm. The work you do during this phase is dependent on your body weight (1.5Watt/kg) and thus kept constant. After cool down and a short recovery period, you will receive two questionnaires.

T3: Duration 90min [45min time trial]

After warm up (10 min) a 45 min time trial begins. You will cycle at a constant 95 rpm with the goal to achieve the highest possible performance over time. During the time trial, a drop of blood will be taken from your earlobe for the lactate analyses every 10 minutes. After cool down and a short recovery period, you will receive two questionnaires.

T4: Duration 90min [45min time trial]

At the 4th test day you will receive either BCAAs, a placebo or nothing at the beginning of the test, depending on which group you were allocated to. As this is a scientific study, neither you, not the experimenter knows which experimental group (BCAA, placebo or control) you are in. The rest of the time trial is identical to the process of test day 3 (T3) (see above).

The time trials on test day 3 and 4 take place at the same time of day (in the morning either 8 or 10 am) and at the same day of the week with exactly 1 week in between, to minimize influences from natural performance variations. Please come fasted (no breakfast, no coffee) on the last two test days.

Risks and side effects

The tests will be carried out by medically trained professionals. The spiro ergometry does not have risks or side effects. In rare cases, the electrode, which are attached to the skin to measure heart function could cause a short lasting local reddening of the skin. The lactate diagnostics at the ear lobe can cause a small hematoma (bruise) at the ear lobe, which will disappear within a few days.

The nutritional supplement tested in this study consists of branched chain amino acids (BCAA), which are well compatible. Only in the case of severe over dosage, they can cause slight nausea or diarrhea. Neither the nutritional supplement nor the test itself causes an impairment in the ability to drive.

**Voluntariness of participation**

Participation in this study is voluntary and you may quit participation at any point without any disadvantages.

**Benefit of the study**

This study investigates the effect of BCAAs on endurance performance in athletes. After study completion, you will receive the results of the performance assessment, which you may use as a performance indicator for your training plans.

Data protection

Those who have direct contact to participant specific data must adhere to professional discretion. All information we receive in the context of this study will be treated confidentially. During analysis and processing of your data, your records are made anonymous, so that no references to you or your family are possible.

Participants insurance

For the protection of the participants an insurance was taken up with ECCLESIA mildenberger HOSPITAL GmbH insuring the way to and from the laboratory and the time spent at the laboratory.

Expense allowance

For study participation, you will receive an expense allowance of 40 € and the results of your lactate and spirometry diagnostics which have a value of 250 €.

**Questions and interests regarding study outcomes**

To ensure neutrality for all participants, we can provide further information only after study completion. If you provide your email address we will inform you about study outcomes. You will receive your personal results of the performance test after finishing the study.

Kind regards,

Ellen K. Broelz, MSc (experimenter)

**Informed consent regarding the participation in the study**

**„Acute effect of a nutritional supplement on endurance performance – a placebo controlled double blind study“**

Herewith I declare, that I received, read and agree to the information sheet regarding the study „Acute effect of a nutritional supplement on endurance performance – a placebo controlled double blind study“.

All my questions regarding the study protocol were answered by the experimenter. I had enough time for my decision to participate in the study and I know, that my participation is voluntary. I was informed, that I may retract my consent at any time without indication of reasons and without any disadvantages for me.

I know, that my data will be stored in anonymous form and will be used only for scientific purposes.

With my signature, I declare my consent to participate in the study.

**I consent to the transfer of the data collected about me in the above described form, considering professional discretion and data protection. I can approach the experimenter and look at my data at any time.**

_______________ _________________ _________________________________

Tübingen, date Signature Name of participant

_______________ ______________­­­­­____ _________________________________

Tübingen, date Signature Name of experimenter
