# Supplementary material for: Increasing effort without noticing: A randomized controlled pilot study about the ergogenic placebo effect in endurance athletes and the role of supplement salience
Source: PLoS One. 2018 Jun 11;13(6):e0198388. doi: 10.1371/journal.pone.0198388 (PMC5995445; doi:10.1371/journal.pone.0198388)
Supplement: S5 File — (DOCX) [file pone.0198388.s005.docx]

Medizinische Fakultät

Medizinische Klinik V

Abteilung Sportmedizin

Univ.-Prof. Dr. med. A. Nieß

Ärztlicher Direktor

Lehrstuhl für Sportmedizin

Telefon +49 7071 29 - 86 493

Telefax +49 7071 29 - 25 028

andreas.niess@med.uni-tuebingen.de

Universitätsklinikum Tübingen · Medizinische Klinik V (Sportmedizin)

Hoppe-Seyler-Str. 6 **^.^** 72076 Tübingen

**Ansprechpartner:**

Ellen K. Broelz

ellen.broelz@med.uni-tuebingen.de

Mobil: 0178 2178 010

**Aufklärungsbogen zur Studie: „Akutwirkung eines Nahrungsergänzungsmittels auf die Ausdauerleistung - eine placebokontrollierte Doppelblindstudie“**

Lieber Studieninteressent,

wir freuen uns über Ihr Interesse an unserer Studie zum Thema „Akutwirkung eines Nahrungs­ergänzungsmittels auf die Ausdauerleistung - eine placebokontrollierte Doppelblind­studie“ teilzunehmen. In dieser Studie soll gezeigt werden, dass verzweigtkettige Aminosäuren (Branched Chain Amino Acids), welche für ihre unterstützende Wirkung in der Regenerationsphase bekannt sind, eine kurzfristige Steigerung der Ausdauerleistung erzeugen können. Die leistungssteigernde Wirkung von BCAAs ist sowohl auf zentrale, als auch auf periphere Prozesse zurückzuführen. Bislang gibt es nur wenige Studien, welche den akuten Effekt dieser Aminosäuren im Ausdauerbereich untersuchen. Diese Studie wird von Frau E. Broelz, MSc der Abteilung Sportmedizin des Universitätsklinikums Tübingen unter der Leitung von Prof. Dr. med. A. Nieß und Dipl.-Sportwiss. P. Schneeweiß durch­geführt.

Studienteilnahme

Wir suchen gesunde, ambitionierte, männliche Ausdauersportler aus den Bereichen Radsport und Triathlon im Alter von 18 bis 40 Jahren. Sie sollten leistungs­orientiert 3-5 Mal pro Woche auf dem Rad trainieren und regelmäßig an Wettkämpfen teilnehmen.

Zum Zeitpunkt der Untersuchung sollten keine schwerwiegenden Erkrankungen vorliegen oder Medikamente eingenommen werden. Des Weiteren sollte in den 4 Wochen vor Studien­beginn keine Trainingspause liegen. Falls dies dennoch der Fall ist, informieren Sie uns bitte.

Studienablauf

Studiendauer und -ort

Die Studie wird von Februar bis Juni 2014 ablaufen und an jeweils 4 Messtagen innerhalb von 2-4 Wochen durchgeführt. Die Messungen finden im Ergometrielabor der Sportmedizin Tübingen statt. Es ist für diese Studie wichtig, dass Sie an Messtag 3 und 4 zu den Time-Trials nüchtern erscheinen, d.h. 12 h keine Nahrung sowie Kaffee bzw. Koffeinhaltiges zu sich nehmen.


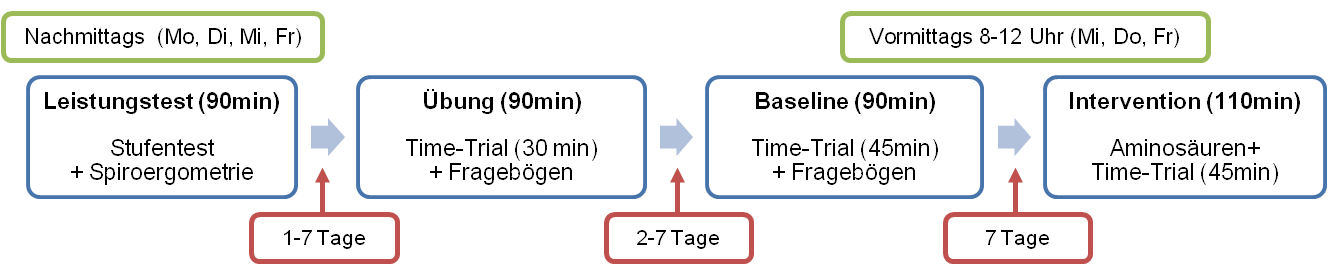
Übersicht

Zunächst bitten wir Sie, den Aufklärungsbogen zu lesen und bei Einverständnis zur Teilnahme diesen zu unterschreiben. Danach werden Sie per Zufallsprinzip der BCAA-, Placebo- oder der Kontrollgruppe zugeordnet. Je nach Gruppe erhalten Sie am 4. Messtag ein BCAA-Präparat, ein Placebo-Präparat oder keine Intervention.

Allgemein

Zu allen Messtagen sollten Sie in ihrer regulären Radbekleidung inkl. Schuhen erscheinen. Vor Ort gibt es Umkleide- und Duschmöglichkeiten. **Wichtig:** Bitte bringen Sie ihre eigenen Pedale (passend zu Ihren Schuhen) mit.

Leistungserfassung

T1: Dauer ca. 90min [Leistungsdiagnostik]

Zu Beginn der Studie werden Ruhe-EKG, Körpergröße und Gewicht gemessen. Anschließend werden Sie von einem Prüfarzt kurz untersucht, welcher Sie dann für die Studie zulässt. Danach beginnt die Leistungs­diagnostik. Sie werden auf einem Fahrradergometer nach einem klassischen Stufen­protokoll belastet. Die Sitzposition auf dem Fahrradergometer können Sie selbst aus­wählen. Finden Sie daher vor Protokollbeginn Ihre präferierte Einstellung. Das Protokoll läuft wie folgt ab: Sie starten bei einer Leistung von 40 Watt. Alle drei Minuten steigert sich die Intensität um 40 Watt. Sie absolvieren den Test so lange, bis sie erschöpft sind oder andere körperliche Beschwerden (z.B. Gelenkschmerz) auftreten. Den Zeitpunkt des Abbruchs können Sie selbständig zu jeder Zeit festlegen. Gleichzeitig werden Belastungs-EKG, Laktatleistungsdiagnostik und eine Spiroergometrie durchgeführt. Bei der Spiroergometrie haben Sie eine Atemmaske auf, um Ihre Atemgase zu messen, die zur Objektivierung der kardiopulmonalen Leistungsfähigkeit dienen. Bei der Laktatleistungsdiagnostik wird am Ende jeder Stufe und nach Belastung ein Tropfen Blut (20µl) aus Ihrem Ohr­läppchen entnommen.

T2: Dauer ca. 90min [Probe Time-Trial (Dauer: 30min)]

Am zweiten Messtag absolvieren Sie einen 30 minütigen Probe Time-Trial, um sich an die isokinetische Belastung und das SRM-Fahrradergometer zu gewöhnen. Sie fahren bei konstanter Trittfrequenz (95 U/min) mit dem Ziel, Ihre persönlich bestmögliche Leistung zu erzielen. Während dem 10-minütigen Einfahren und Ausfahren können Sie Ihre Trittfrequenz zwischen 70 und 100 U/min frei wählen. Die in diesen Abschnitten zu erbringende Leistung ist an Ihr Körpergewicht (1,5 Watt/kg) gekoppelt und somit konstant. Nach Ausfahren und kurzer Erholung füllen Sie noch zwei Fragebögen aus.

T3: Dauer ca. 90min [45min Time-Trial]

Nach der Einfahrphase (10 min) beginnt ein 45 minütiger Time-Trial. Sie fahren hierfür bei konstanten 95 U/min mit dem Ziel über den gesamten Zeitraum die höchst mögliche Leistung zu erzielen. Während des Time-Trials wird alle 10 Minuten ein Tropfen Blut (20 µl) für die Laktatdiagnostik aus dem Ohrläppchen entnommen. Nach Ausfahren und kurzer Erholung füllen Sie noch zwei Fragebögen aus.

T4: Dauer ca. 110min [45 min Time-Trial]

Am vierten Messtag erhalten Sie vor Testbeginn entweder ein BCAA-Präparat, ein wirkstoff­freies Placebo-Präparat oder kein Präparat, je nach dem welcher Versuchsgruppe Sie zugeordnet wurden. Da es sich hier um eine wissenschaftliche Untersuchung handelt, wissen weder Sie, noch Ihr Versuchsleiter welcher Versuchsgruppe (BCAA, Placebo oder Kontrolle) Sie zugeordnet sind. Der Ablauf des Time-Trials läuft danach identisch mit dem an Messtag 3 (T3) ab (siehe oben).

Die Time-Trials an den Messtagen 3 & 4 finden zur gleichen Tageszeit (Vormittags um 8:00 oder 10:00 Uhr) und am gleichen Wochentag im Abstand von exakt einer Woche statt, um Einflüsse natürlicher Leistungsschwankungen zu minimieren. Wir bitten Sie an beiden Messtagen nüchtern im Labor zu erscheinen (kein Frühstück, kein Kaffee).

Risiken und Nebenwirkungen

Die Untersuchungen werden von medizinisch geschultem Fachpersonal durchgeführt. Die Spiroergometrie ist mit keinen Risiken oder Nebenwirkungen verbunden. Die Elektroden, die zur Erfassung der Herzfunktion auf die Haut geklebt werden können in seltenen Fällen eine kurzfristige Hautrötung verursachen. Durch die Laktat-Bestimmung am Ohrläppchen kann eine Rötung oder ein kleines Hämatom (blauer Fleck) am Ohrläppchen entstehen, welches sich jedoch binnen weniger Tage zurückbildet.

Bei dem hier eingesetzten Nahrungsergänzungsmittel handelt es sich um verzweigkettige Aminosäuren, welche sehr gut verträglich sind. Nur im Falle einer extremen Über­dosierung kann es zu Beschwerden wie leichte Übelkeit und Durchfall kommen. Weder die Unter­suchung noch das verwendete Nahrungsergänzungsmittel ziehen eine Beeinträchtigung der Verkehrstüchtigkeit nach sich.

Freiwilligkeit der Teilnahme

Die Teilnahme an dieser Studie ist freiwillig und Sie können die Teilnahme jederzeit beenden, ohne dass Ihnen dadurch Nachteile entstehen.

Nutzen der Studie

Diese Studie untersucht die Wirkung von BCAAs auf die Ausdauerleistungen bei Sportlern. Sie erhalten nach Studienabschluss alle Ergebnisse der Leistungsdiagnostik, welche Sie als Leistungsmaß und zur weiteren Trainingsplanung nutzen können.

Datenschutz

Diejenigen Mitarbeiter, die durch den direkten Kontakt mit Ihnen über personenbezogene Daten verfügen, stehen unter Schweigepflicht. Alle Informationen, die wir im Rahmen des Projekts von Ihnen erhalten, werden absolut vertraulich behandelt. Bei der Auswertung und Aufbereitung Ihrer Daten werden wir die Unterlagen pseudonymisieren, so dass keine Rück­schlüsse auf Sie oder Ihre Familie gezogen werden können.

Probandenversicherung

Zum Schutz der Probanden wurde über die ECCLESIA mildenberger HOSPITAL GmbH eine Versicherung für den Weg zum/vom Labor und die Zeit des Aufenthaltes im Labor ab­ge­schlossen (sogenannte Wege-Unfall-Versicherung).

Aufwandsentschädigung

Sie erhalten für die Studienteilnahme eine Aufwandsentschädigung von 40€, sowie die Ergebnisse Ihrer Laktatleistungsdiagnostik und Spiroergometrie im Regelfall Wert von ca. 250,- Euro.

Fragen und Interesse bzgl. der Studienergebnisse

Aufgrund der sicherzustellenden Neutralität für jeden Probanden können wir erst nach Ab­schluss der Studie darüber Auskunft geben. Bei Angabe der Email-Adresse werden wir aber jeden Interessenten über die Studiengesamtergebnisse informieren. Ihre persönlichen Ergebnisse der Leistungsdiagnostik erhalten Sie nach dem Abschluss der Studie.

Mit freundlichen Grüßen

Ellen K. Broelz, MSc (Studienleitung)

**Einverständniserklärung zur Teilnahme an der Studie:**

**„Akutwirkung eines Nahrungsergänzungsmittels auf die Ausdauer­leistung - eine placebokontrollierte Doppelblind­studie“**

Hiermit bestätige ich, dass ich das Informationsblatt zur Studie „Akutwirkung eines Nahrungs­ergänzungsmittels auf die Ausdauerleistung - eine placebokontrollierte Doppel­blindstudie“ erhalten, gelesen und verstanden habe.

Alle meiner Fragen bezüglich des Studienablaufes wurden von der Versuchsleiterin beantwortet. Ich hatte ausreichend Zeit, mich zur Teilnahme an der Studie zu entscheiden und weiß, dass die Teilnahme freiwillig ist. Ich wurde darüber informiert, dass ich jederzeit und ohne Angabe von Gründen diese Zustimmung widerrufen kann, ohne dass dadurch Nachteile für mich entstehen.

Mir ist bekannt, dass meine Daten anonym gespeichert und ausschließlich für wissen­schaftliche Zwecke verwendet werden.

Mit meiner Unterschrift erkläre ich mich einverstanden an dieser Studie teilzunehmen.

**Mit einer Weitergabe der über mich erhobenen Daten in oben beschriebener Art und Weise bin ich unter Berücksichtigung der Schweigepflicht und des Datenschutzes einverstanden. Ich kann jederzeit meine Daten beim Studienleiter einsehen.**

_______________ _________________ ________________________________

Tübingen, den Unterschrift Name des Probanden in Blockschrift

_______________ _________________ ________________________________

Tübingen, den Unterschrift Name des aufklärenden Wissenschaftlers

in Blockschrift
